# The quality of care for type 2 diabetes mellitus management in Malaysian primary health care settings: A scoping review of ABC (glycated haemoglobin A1c, blood pressure, and LDL-cholesterol)

**DOI:** 10.1371/journal.pone.0355227

**Published:** 2026-07-31

**Authors:** Mohamad Zulfikrie Abas, Norshahiratul Atiqah Mohd Zaidi, Pei Jia Lee, Xin Rou Teh, Kim Sui Wan, Azah Abdul Samad, Huan Keat Chan, Sheamini Sivasampu, Swee Hung Ang

**Affiliations:** 1 Institute for Clinical Research, National Institutes of Health, Ministry of Health Malaysia, Shah Alam, Selangor, Malaysia; 2 Institute for Public Health, National Institutes of Health, Ministry of Health Malaysia, Shah Alam, Selangor, Malaysia; 3 Primer Health Branch, Family Health Development Division, Ministry of Health Malaysia, Putrajaya, Malaysia; University of Benghazi, LIBYA

## Abstract

**Background:**

Diabetes is a major health concern in Malaysia, yet no comprehensive review has assessed the quality of care for these patients. This study aims to systematically review published evidence on type 2 diabetes mellitus (T2DM) management in Malaysian primary health care (PHC), focusing on the achievement of glycated haemoglobin (HbA1c), blood pressure (BP), and LDL-cholesterol (LDL-C) targets (ABC control).

**Methods:**

A scoping review was conducted, involving a comprehensive search of four databases (PubMed, Embase, Scopus, and MyMedR) and grey literature for publications up to December 2024. Studies were included if they reported on at least one ABC indicator among the general adult T2DM population in Malaysian PHC settings. The scoping review followed the Joanna Briggs Institute (JBI) methodology and PRISMA-ScR guidelines. EndNote was used for deduplication, and Rayyan was utilised for the screening process. Data were extracted and synthesised narratively.

**Results:**

A total of 109 publications were included. Publications increased post-2010 but remained geographically concentrated in urban states. Large-scale studies heavily relied on the National Diabetes Registry. HbA1c was the most reported indicator. Findings revealed no evidence of improvement in HbA1c and BP control over two decades; achievement rates were 30–45% for HbA1c (<7.0%) and 20–50% for combined BP target (<130/80 mmHg). Conversely, LDL-C control (≤2.6 mmol/L) showed a modest improvement, with achievement rates rising from approximately 30% to 50% over a decade.

**Conclusion:**

Despite a substantial increase in research, the HbA1c and BP control for T2DM in Malaysian PHC has remained static and suboptimal, highlighting a persistent gap between clinical guidelines and real-world outcomes. The modest improvement in LDL-C suggests that progress is achievable. These findings underscore the need for a balanced policy focus on all three ABC indicators, strategies to overcome systemic barriers, and continued investment in the national registry to guide evidence-based improvements in diabetes care.

## Introduction

Diabetes mellitus (DM) is a critical global public health challenge [1]. In 2024, approximately 11.1% adults (589 million adults) were living with the condition, and this figure is projected to reach 13.0% (852.5 million adults) by 2050 [2]. Malaysia, a Southeast Asian nation comprising 13 states and three federal territories, mirrors this alarming trend. The country reported a national DM prevalence of 15.6% in 2023, which is largely driven by rapid urbanisation, population ageing, and lifestyle shifts [2,3]. In Malaysia, the public primary health care (PHC) system serves as the essential cornerstone for chronic disease management across these regions and bears the primary responsibility for the diagnosis, treatment, and long-term care of patients with diabetes [4]. Effective management centres on three key clinical indicators: glycated haemoglobin (HbA1c), blood pressure (BP), and low-density lipoprotein cholesterol (LDL-C), collectively known as ABC control. These indicators are widely recognised as essential proxies for evaluating the quality of diabetes care and are critical for reducing long-term microvascular and macrovascular complications [5,6].

Despite the existence of established clinical practice guidelines, national data from the National Health and Morbidity Survey (NHMS) and the National Diabetes Registry (NDR) consistently indicate that many patients in Malaysian PHC settings fail to achieve optimal ABC targets [3,7,8]. While numerous studies have addressed specific aspects of diabetes control, these existing publications typically report findings from isolated cohorts or specific registry datasets. For example, research has ranged from multi-centre clinical audits such as the Malaysian DiabCare studies to various population-based longitudinal analyses and localised medical record reviews [8,9]. To date, however, there has been no comprehensive synthesis that integrates these disparate findings to map the broader landscape of collective ABC control in Malaysia. Thus, a significant gap exists in understanding the extent and characteristics of existing research, particularly regarding the reported indicators and the various clinical targets utilised across different studies. This lack of synthesis obscures the ability to identify established patterns and detect longitudinal trends in control levels across the years, which may help in recognising common systemic failures across the PHC network.

To address these gaps, this scoping review aims to systematically map and synthesise the published evidence on the quality of T2DM care within Malaysian PHC settings. The review is guided by two research questions:

i What are the characteristics of studies reporting estimates of ABC control among patients with T2DM in Malaysian PHC settings?
ii What are the reported measures and estimates of ABC control among patients with T2DM treated in PHC settings in Malaysia?

Given the observational nature of these questions and the expected variability in study designs and outcome reporting, a scoping review was deemed the most appropriate methodological approach. By mapping the nature, range, and extent of research activity, this review specifically examines the characteristics of the evidence base, the heterogeneity in clinical target definitions, and the trends in control achievement to clarify the current state of diabetes care quality research. Ultimately, this synthesis intends to identify knowledge gaps, inform future research directions, and support the development of targeted health policies aimed at bridging the gap between clinical guidelines and real-world outcomes in Malaysia.

## Methods

The scoping review was conducted in accordance with the methodological guidance of the Joanna Briggs Institute (JBI) for scoping reviews [10]. Reporting adhered to the Preferred Reporting Items for Systematic Reviews and Meta-Analyses extension for Scoping Reviews (PRISMA-ScR) [11], ensuring methodological rigour, transparency, and reproducibility.

### Eligibility criteria

The eligibility criteria were defined using the Population, Concept, and Context (PCC) framework, as recommended by the JBI manual for scoping reviews [10]. This review was conducted as part of a larger scoping review (MyABCMap) that aimed to systematically map the literature on the quality of care for adult patients with T2DM, hypertension, or dyslipidaemia in Malaysia.

#### Population.

This review included studies involving adult patients (aged 18 years and above) diagnosed with T2DM. To ensure comparability and relevance to system-wide diabetes care delivery, only studies reporting outcomes for the general adult T2DM population were included. Studies that focused exclusively on restricted subpopulations, such as older adults only, women only, or patients with specific isolated comorbidities like T2DM with chronic kidney disease, were excluded. This restriction was applied to avoid selection bias and overrepresentation of subgroup-specific outcomes that may not reflect the broader quality of care provided at the PHC level. However, we acknowledge that while this approach is essential for a system-level assessment, it may potentially underestimate specific inequities in care quality existing within these distinct subgroups.

#### Concept.

The primary concept was the mapping of the quality of diabetes care as measured by three key clinical indicators: HbA1c, BP, and LDL-C, collectively referred to as ABC control. Studies were included if they reported at least one of these indicators. These clinical targets were used as surrogate markers to reflect the quality of diabetes care provided, rather than to assess downstream clinical outcomes such as diabetes-related complications or mortality.

#### Context.

The review was restricted to studies and programmes conducted within the PHC setting in Malaysia. This included PHC facilities across the public and private sectors, such as Ministry of Health (MOH) health clinics, private general practitioner clinics, and primary care services operated by universities.

### Types of evidence sources

The review included peer-reviewed research articles employing any methodological approach (quantitative, qualitative, or mixed-methods) and technical reports published by the MOH Malaysia, specifically from the Family Health Development Division and the Disease Control Division. Guidelines, book chapters, literature reviews, commentaries, editorials, conference abstracts, and letters to the editors were excluded.

### Search methods

A comprehensive search was conducted across four key databases, which included PubMed, Embase, Scopus, and the Malaysian Medical Repository (MyMedR), to identify relevant studies. To ensure thoroughness and minimise the risk of missing pertinent literature, backward citation tracking (snowballing) of the reference lists of all included articles was also performed. In addition to database searches, a manual search was undertaken to identify relevant grey literature, primarily consisting of reports published by the MOH Malaysia. These strategies were designed to capture a broad scope of relevant literature, encompassing both peer-reviewed studies and grey literature, published from the inception of each database up to 31st December 2024.

The search strategy combined relevant keywords and Medical Subject Headings (MeSH) terms, tailored to capture studies related to T2DM and PHC settings in Malaysia. Customised search strings were developed for each selected database to ensure sensitivity and relevance. The full search strategies for all databases are provided in the S1 Table.

### Selection process

All titles and abstracts identified through the database searches were imported into EndNote reference management software. Duplicate records were removed using the “Find Duplicates” function in EndNote. After deduplication, the remaining citations were exported and uploaded into Rayyan, an online screening tool, for the selection process.

The screening and assessment process was applied uniformly to both peer-reviewed articles and grey literature to ensure consistency in the quality of the evidence mapping. Two reviewers independently screened the titles and abstracts against the predefined inclusion and exclusion criteria. For grey literature, such as MOH reports that may not have standard abstracts, the table of contents and executive summaries were reviewed using the same eligibility framework. Any disagreements were resolved through discussion, and when necessary, the opinion of a third reviewer was sought. Reasons for exclusion at this stage were documented.

Full-text articles and complete grey literature reports of potentially eligible studies were then retrieved and uploaded into EndNote for the second stage of the screening process. For reports that were not immediately available, reviewers attempted to retrieve full texts through institutional library services, searches for preprints or archived versions on academic networking sites, and at least two separate attempts of direct requests to corresponding authors via email or academic networking platform (ResearchGate). Reports were categorised as ‘not retrieved’ only after all these avenues were exhausted or if the author’s contact information could not be traced. A detailed log of these retrieval attempts and the reassessment of excluded records is provided in S2 Table. The full-text screening followed the same rigorous procedure as the initial screening, with two reviewers independently assessing each document for final inclusion. Reviewers were not blinded to the journal name, authorship, or institutional affiliations during the selection process.

### Data charting process

Two reviewers jointly developed a standardised data extraction form to identify and define the variables for extraction, accompanied by a detailed instruction manual to ensure consistency and accuracy during the process. Before the formal review, a pilot test of the form was conducted to assess its clarity and usability, and revisions were made based on feedback from the pilot phase (S3 Table). The finalised data extraction form was implemented using Google Sheets as the data extraction platform, allowing shared access and real-time updates by the review team. Two reviewers then independently extracted data from the included studies. Any discrepancies were resolved through discussion and consensus. When agreement could not be reached, a third reviewer was consulted to facilitate a final decision.

Data were extracted on key article characteristics, including author(s), year of publication, study design, sample size, and study location. Information extracted for the ABC parameters included the proportion of patients meeting target values and the mean or median values, where available. The complete list of extracted data items is presented in the S4 Table. Information regarding funding sources of the included articles was attached in S5 and S6 Tables. The PRISMA-ScR checklist was attached in S7 Table.

### Data synthesis

The extracted data were synthesised and presented using a narrative approach, structured around the scoping review’s research questions. In studies where results were reported separately for subgroups (e.g., by gender), the subgroup data were aggregated to generate a single estimate representing the overall T2DM population of the study. Descriptive numerical summaries, including frequencies and percentages, were used to describe the characteristics of the included studies, such as publication year, study design, geographical distribution, and sample size.

To visualise the findings, a series of charts and tables was generated. These included heatmaps to illustrate the distribution of studies over time and across states, and scatter plots to present the reported estimates for HbA1c, BP, and LDL-C outcomes. This approach allowed for a comprehensive mapping of the existing literature and a clear presentation of the quality of care estimates. In line with scoping review methodology, a formal meta-analysis was not conducted due to the anticipated heterogeneity in study designs, populations, and outcome definitions.

### Protocol and registration

The review protocol for the overarching scoping review was registered on Open Science Framework (OSF) with the associated project: osf.io/gh7b4 and registration DOI: https://doi.org/10.17605/OSF.IO/7GNYM

## Results

### Search and selection of studies

**Fig 1:**
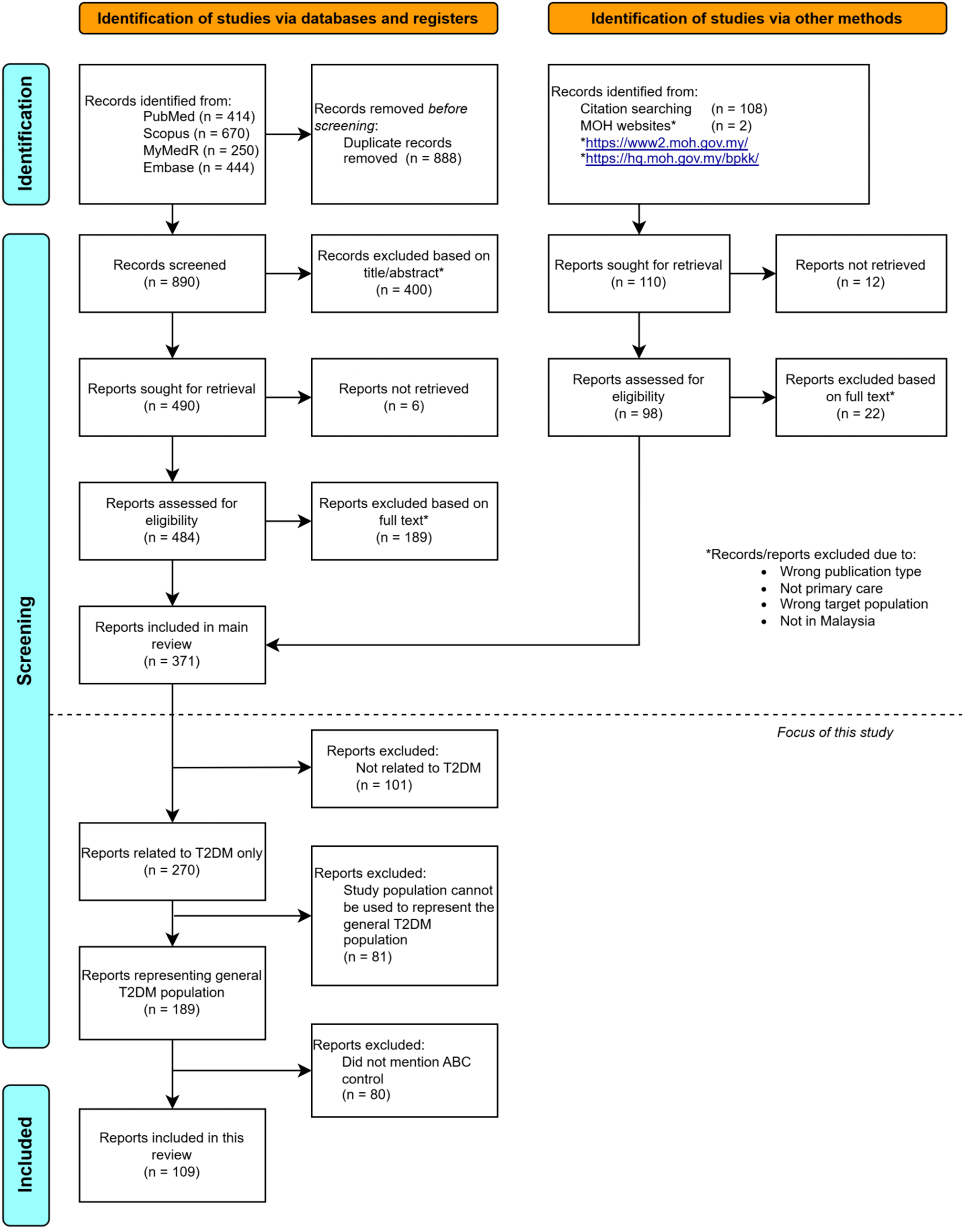
PRISMA Flow chart for the articles included in the scoping review.

The initial database search yielded 1888 records. After removing duplicates, 1000 publications were screened based on their titles and abstracts. This resulted in 582 full-text publications being assessed for eligibility. Following a detailed screening and selection process, a final total of 109 publications met the inclusion criteria and were included in this scoping review (S4 Table). Fig 1 illustrates the PRISMA flow diagram, which outlines the study selection process. The selection process identified a substantial and diverse body of evidence, providing a comprehensive basis for mapping the T2DM quality of care landscape in Malaysia across more than two decades.

**Fig 2:**
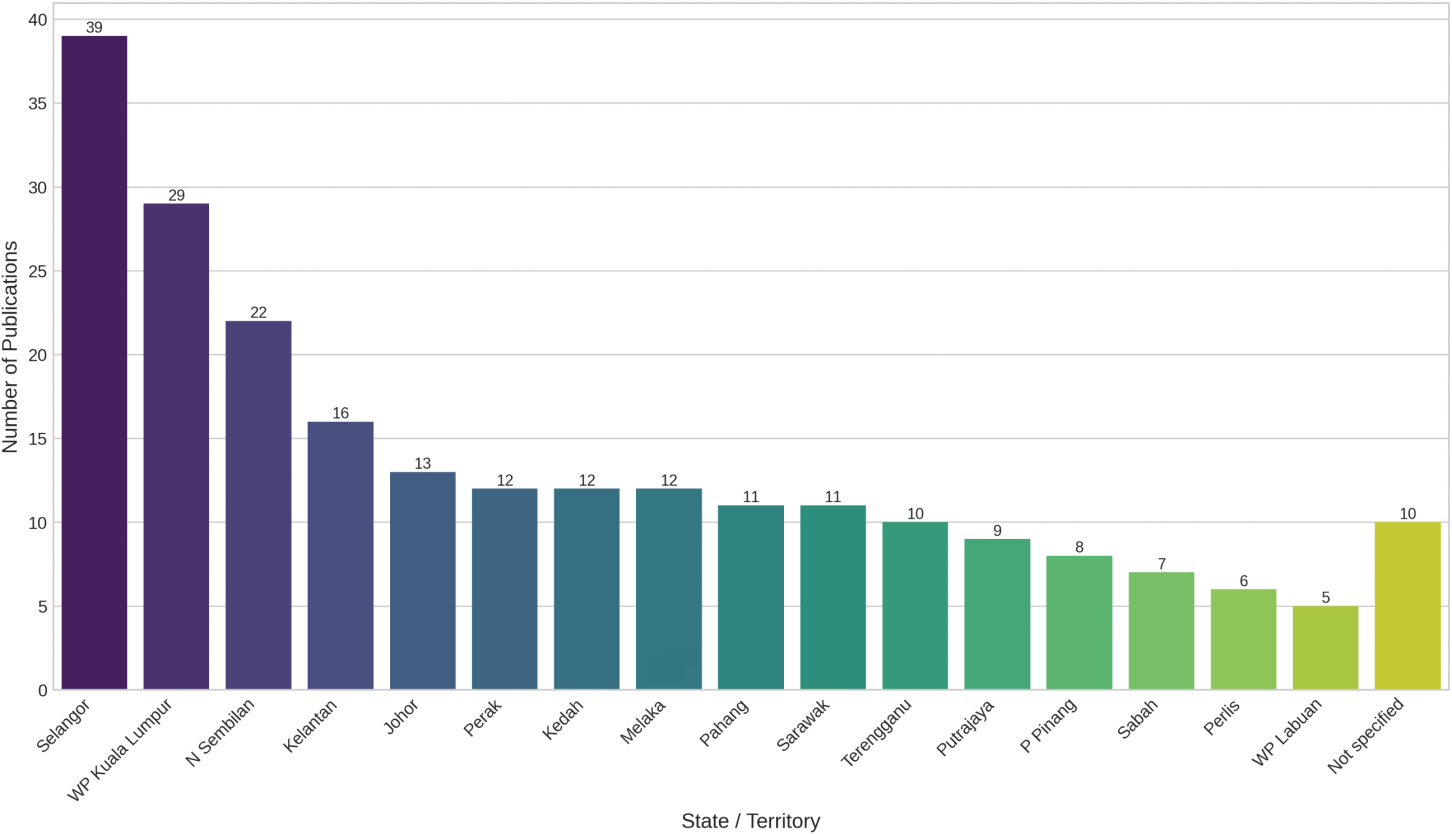
Distribution of study sites for the 109 publications by state/territory.

**Fig 3:**
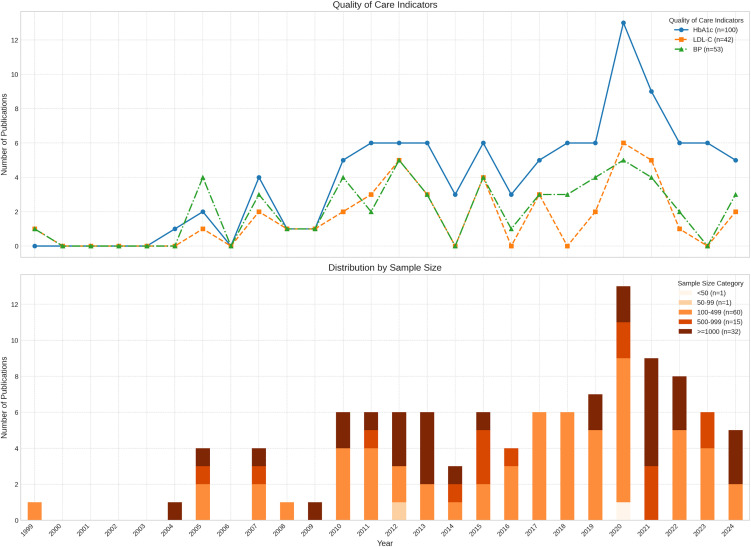
Evidence map of characteristics of publications reporting on the ABC estimates among T2DM population in Malaysian PHC setting (N = 109).

**Fig 4:**
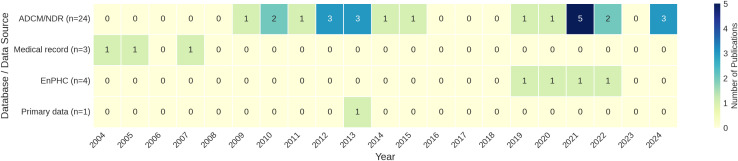
Distribution of data sources utilised in publications with sample size of at least 1000 (N = 32).

**Fig 5:**
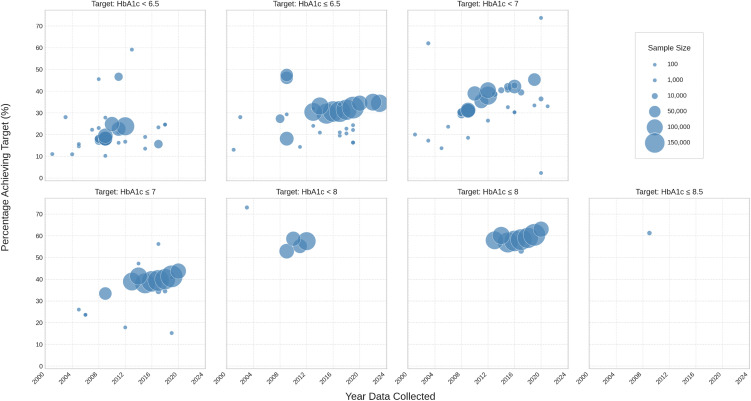
Evidence map of reported HbA1c target achievement estimates across publications.

**Fig 6:**
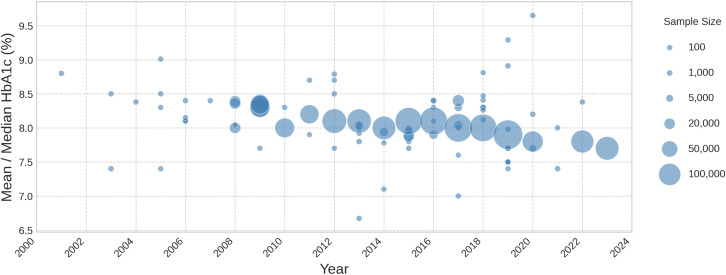
Evidence map of reported HbA1c estimates across publications (N = 95).

**Fig 7:**
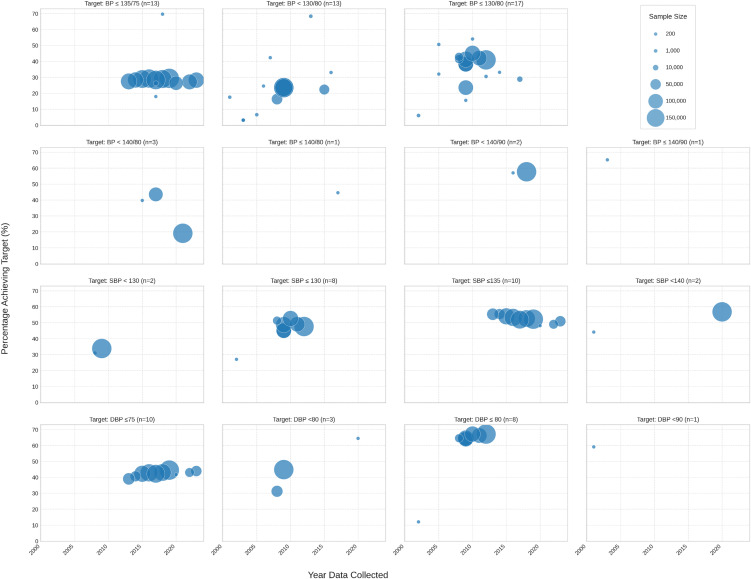
Evidence map of reported BP target achievement estimates across publications.

**Fig 8:**
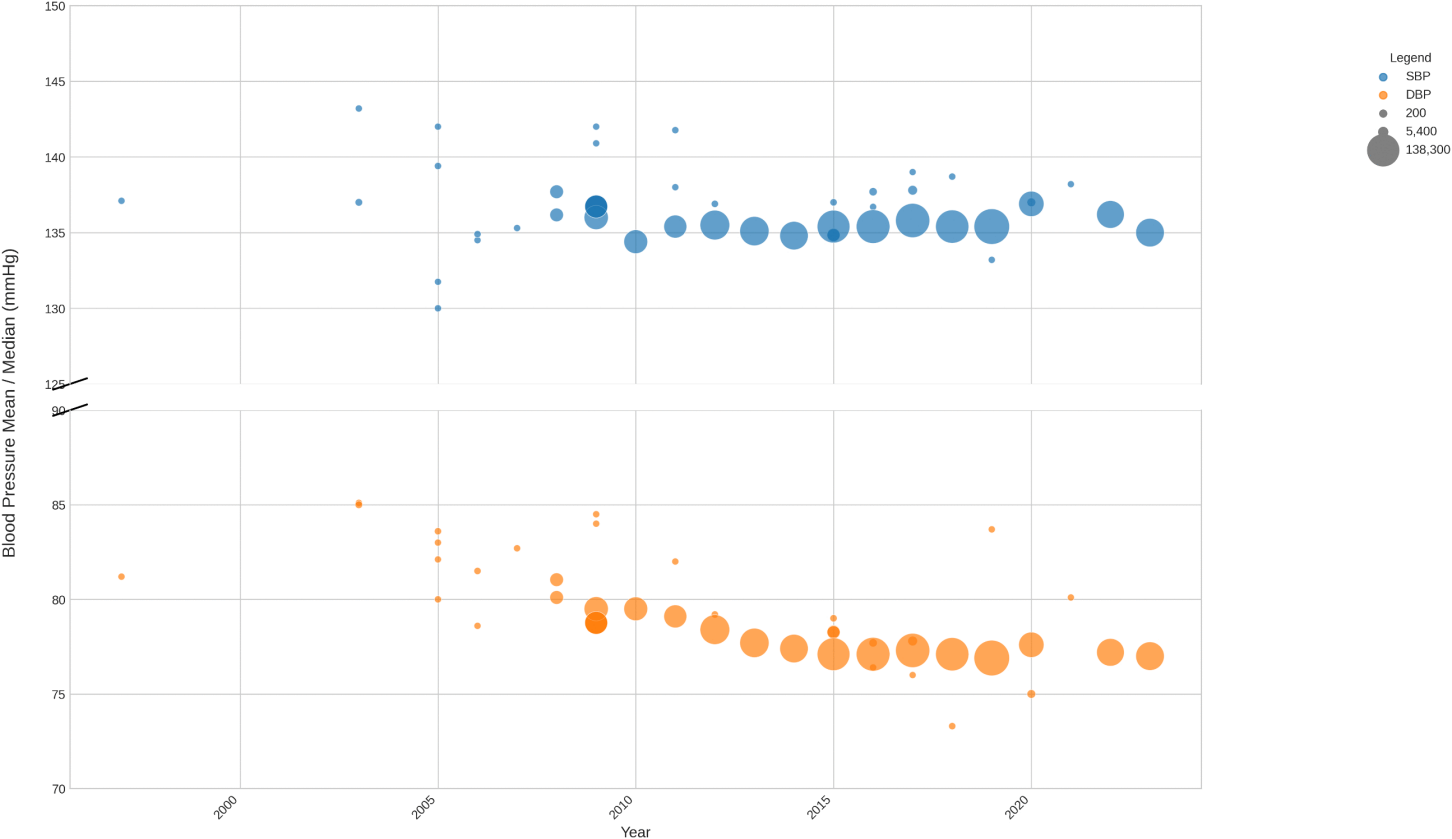
Evidence map of reported SBP and DBP estimates across publications (N = 46).

**Fig 9:**
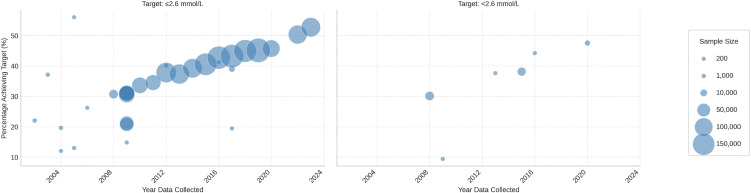
Evidence map of reported LDL-C target achievement estimates across publications.

**Fig 10:**
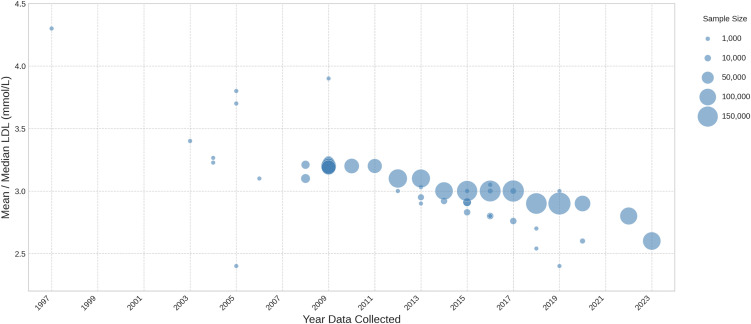
Evidence map of reported LDL-C estimates across publications (N = 47).

The subsequent evidence map (Figs 2–10) presents information and estimates extracted from these publications, providing a comprehensive visualisation of the reported outcomes to illustrate the landscape of available research. When interpreting these maps, it should be noted that the counts presented may exceed the total number of included publications. This occurs because some studies reported data from multiple sites across different states, while others reported outcomes across multiple clinical target categories, including more than one HbA1c threshold; the same applies to BP and LDL-C indicators.

### Characteristics of studies reporting estimates of ABC control

#### Temporal and geographical distribution.

The 109 publications included were published over 26 years, from 1999 to 2024. The volume of publications on this topic has increased substantially over time, with a notable acceleration in publications from 2010 onwards (S1 Fig). This increase in volume suggests a growing academic and policy interest in diabetes care quality, yet the geographical distribution remains disproportionately concentrated. As detailed in Fig 2, the state of Selangor (39 publications), Wilayah Persekutuan Kuala Lumpur (29), and Negeri Sembilan (22) dominate the literature. In contrast, rural or less urbanised states such as Johor (13), Kedah (12), and Sarawak (11) are underrepresented, with several territories having fewer than ten publications each. While research output has increased significantly in recent years, a clear geographical disparity persists leaving a significant data gap regarding the quality of care in rural and underserved regions.

#### Profile of included publications.

The profile of the included publications reveals a research landscape characterised by a predominant focus on glycaemic control. As shown in Fig 3, HbA1c was reported in 91.7% (n = 100) of studies, nearly double the reporting frequency of BP (48.6%, n = 53) and LDL-C (48.5%, n = 42). Methodologically, the data sources for large-scale publications with sample sizes of 1000 or more has transitioned from localized manual medical record reviews toward a heavy reliance on the Malaysian NDR or its predecessor, the Adult Diabetes Control and Management (ADCM) database, particularly since 2009 (Fig 4). These registry-based studies account for 75% of all large-scale publications

### Reported Estimates of Quality of Care (ABC Control)

#### A: HbA1c control.

The mapping of HbA1c control outcomes reveals a continuing gap between clinical guidelines and real-world achievement. Despite considerable heterogeneity in target definitions (Fig 5 and S2 Fig), ranging from <6.5% to ≤8.5%, a consistent pattern shows no evidence of clinical improvement. For example, achievement rates for the standard <7.0% target typically show no consistent evidence of improvement, remaining between 30% and 45%. Notably, even when utilising less stringent thresholds (≤8.5%), nearly 40% of patients failed to meet the goal. This trend is further evidenced by the longitudinal mean/median HbA1c values (Fig 6), which have remained relatively stable between 7.5% and 8.5% for two decades without any clear evidence of clinical progress. Overall, glycaemic control in Malaysian PHC illustrates a pattern where, regardless of the target used, achievement rates have remained consistently low and mean/median HbA1c levels have not shown any measurable progress over the last 20 years.

#### B: BP control.

BP control mirrors the trends observed in HbA1c control. While there is significant variation in reported combined and individual BP targets (Fig 7 and S3 Fig), the outcomes consistently fall short of recommended goals. Achievement rates for combined BP targets, typically <130/80 mmHg, largely remain within the 20% to 50% range (Fig 7). Furthermore, mean SBP values have remained relatively stable between 134 mmHg and 138 mmHg over time (Fig 8), indicating that BP control has not seen significant advancements despite evolving clinical guidelines. This lack of measurable progress over time, with achievement rates and mean values remaining consistently outside of optimal ranges, may suggests that systemic barriers to BP management have remained largely unaddressed.

#### C: LDL-C control.

In contrast to the trends in HbA1c and BP, LDL-C management shows a distinct and modest trajectory of improvement. While targets have remained relatively consistent, with 77.4% of studies using ≤2.6 mmol/L (Fig 9 and S4 Fig), the proportion of patients achieving these goals has increased. Specifically, large-scale registry data demonstrate achievement rates rising from approximately 30% in 2009 to around 50% in 2023 based on the ≤ 2.6 mmol/L target (Fig 9). This positive trend is consistent with the longitudinal mean LDL-C values (Fig 10), which show a gradual and visible decrease over time. LDL-C control stands as the only ABC indicator demonstrating a modest trend of improvement, where the increasing achievement rates and decreasing mean values over the past decade suggest that progress in this clinical parameter is achievable within the PHC system.

## Discussion

This scoping review provides the first comprehensive synthesis of the published evidence on the quality of T2DM care in Malaysian PHC settings, as measured by the achievement of ABC control targets. Our findings reveal that despite a substantial expansion of research and national efforts, glycaemic and BP control among patients with T2DM in Malaysian PHC shows no consistent evidence of improvement over the past two decades. We found a substantial increase in research output since 2010, a surge that may be linked to the implementation of Malaysia’s National Strategic Plan for Non-Communicable Diseases (NSP-NCD) 2010–2014 and its subsequent phases, which prioritised NCD management and likely spurred greater monitoring and academic inquiry into care quality [12]. However, this increased research and policy attention has yet to be reflected in significant clinical improvements, pointing to a continuing research–practice gap, where the generation of evidence and policy attention has not been translated into substantial clinical progress.

### Characteristics of the evidence base: geographical distribution and data sources

A key insight from our analysis is that research activity is geographically concentrated in more urbanised states like Selangor and Kuala Lumpur, a reflection of Malaysia’s academic and healthcare infrastructure. These states host many of the country’s major universities and research centres, creating a hub of academic expertise, medical specialists and funding opportunities that make it more feasible to conduct studies. This reflects feasibility and resource availability in more urbanised areas but limits the generalisability of findings to rural and underserved populations, where diabetes care challenges may be even greater [13].

This geographical imbalance carries significant implications for health equity and the interpretation of national care quality. By predominantly focusing on urbanised regions, the existing literature may overlook the unique systemic barriers prevalent in rural and remote PHC settings, such as logistical challenges in medication supply chains, restricted access to specialist support, and varied socioeconomic determinants of health [14,15]. Consequently, the current synthesis might represent a conservative estimate of the national quality gap. The underrepresentation of these underserved populations suggests that the true magnitude of suboptimal diabetes control nationally may be greater than what is currently captured in the published literature, as rural facilities often face more acute resource constraints that hinder the achievement of ABC targets [14,15].

Furthermore, our analysis reveals a heavy reliance on the NDR, which evolved from the earlier ADCM registry [16,17], as the primary data source for most large-scale publications in this area [7,9,18]. This reliance highlights the registry’s central importance as a national tool for health surveillance. This is a matter of practicality and scale; the NDR provides a ready-made, large-scale national dataset, offering researchers an efficient and cost-effective way to conduct powerful statistical analyses that are generalizable to the whole country and important for informing national health policy. While the NDR enables large-scale, standardised research and is invaluable for monitoring diabetes care in Malaysia, this reliance also means that study findings are influenced by the registry’s strengths and inherent limitations, including data coverage, accuracy, and completeness [19].

To address potential concerns regarding selection and reporting bias related to the registry-based publications, it is noteworthy that the quality of Malaysian NDR data is maintained through rigorous control measures implemented by the MOH Malaysia. These include multi-level clinical data audits conducted regularly at the district, state, and national levels, alongside adherence to specific NDR operational guidelines [16,17]. Furthermore, as the NDR data is used to measure diabetes management Key Performance Indicators (KPIs) at the ministerial level, there is a strong institutional mandate for accurate and comprehensive reporting across all participating facilities. These systematic checks suggest that the data quality is robust and that the risk of reporting bias, even across varied PHC settings, is likely minimal. As such, it is imperative to ensure the registry is sustained and its data quality continuously improved, as the value of such national registries in providing the real-world evidence needed to guide health policy is well-established.

Looking forward, the wider adoption and integration of Electronic Medical Records (EMRs) will be crucial. Unlike traditional registries, comprehensive EMR systems can capture rich, longitudinal data on individual patients, offering a far more granular understanding of disease progression and care pathways. However, achieving full, interoperable EMR coverage across the entire Malaysian PHC system will likely require extensive timeline, making the continued support and enhancement of the NDR a critical priority for the medium term.

### The need for a comprehensive approach to ABC control

This scoping review also revealed a higher volume of studies reporting on HbA1c than to BP and LDL-C. This is likely a direct reflection of its established role as a primary KPI for diabetes management within the Malaysian MOH’s quality assurance programs [16,20]. While this focus is important, it may de-emphasise the significance of BP and LDL-C. It is crucial to recognise that BP and LDL-C control are also critical determinants of cardiovascular outcomes. Landmark studies have demonstrated that blood pressure control can have a more profound impact on reducing macrovascular complications than glycaemic control alone [21–23]. Therefore, a predominant focus on HbA1c at the policy and clinical levels might not fully address the primary drivers of cardiovascular risk in this patient population. Adopting a composite KPI that reflects combined ABC control could better incentivise holistic management and align performance evaluation with comprehensive risk reduction. Such a shift would also help close the research–practice gap by translating knowledge about multifactorial management into practice-level accountability.

### Continuing challenges in glycaemic and BP control

Another key finding of this review is the limited evidence of measurable improvement in HbA1c and BP control over time. The majority of studies reported mean/median HbA1c levels above the recommended target of <7.0% for most patients. Similarly, mean/median SBP levels consistently exceeded the common target of <130 mmHg. The proportion of patients achieving these targets remained relatively low, with most studies reporting achievement rates of only 30–45% for HbA1c and 20–50% for combined BP goals. The challenge in achieving glycaemic control is further highlighted by publications using the least stringent targets; nearly 40% of the patients did not achieve an HbA1c threshold of ≤8.5%.

This trend suggests that despite increased attention and monitoring, the underlying systemic, clinical, and patient-level barriers to effective diabetes management in Malaysia have not been sufficiently addressed. These barriers are likely multifactorial and may include clinical inertia [24]; an overburdened primary care system with limited consultation time and resources [4]; and patient-related factors such as low health literacy, financial constraints, and difficulties in adhering to complex lifestyle changes and medication regimens [25–27]. These findings are a point of concern, as suboptimal glycaemic and BP control are major drivers of serious microvascular and macrovascular complications, which impose a significant burden on both patients and the healthcare system.

### Modest improvements in LDL-cholesterol management

Conversely, the data on LDL-C control suggest a modest trend of improvement. This was particularly evident in large-scale publications based on national registry data, which showed a gradual increase in the proportion of patients achieving the LDL-C target of ≤2.6 mmol/L, rising from approximately 30% to 50% over a decade.

Several factors might explain this trend. First, lipid management, primarily through statin therapy, is often pharmacologically simpler than glycaemic or blood pressure control, which frequently requires complex multi-drug regimens and significant lifestyle modifications [5]. Statins are highly effective, generally well-tolerated, and have a more straightforward titration process. Second, national health initiatives and prescribing guidelines may have placed a stronger emphasis on statin use for primary and secondary prevention of cardiovascular disease, leading to better uptake and adherence among clinicians and patients [5,28]. Additionally, the gradual tightening of LDL-C target recommendations over successive Malaysian CPG updates may have motivated more intensive lipid-lowering management, thereby improving achievement rates [5,29–31]. While an achievement rate of approximately 50% remains below optimal levels, this positive trajectory demonstrates that sustained improvement is possible within the Malaysian PHC system and offers valuable lessons that could potentially be applied to improve glycaemic and BP management.

### Implications of target heterogeneity and the lack of individualised target

A significant challenge in interpreting the landscape of diabetes care in Malaysia is the marked heterogeneity in the reporting of clinical targets. For HbA1c, at least four different thresholds were used (e.g., < 6.5%, ≤ 6.5%, < 7.0%, ≤ 7.0%), while the variation in BP targets was even greater. One plausible explanation for this heterogeneity is the evolution of clinical guideline recommendations over time and the use of different reference sources across studies. For instance, successive versions of Malaysia’s T2DM Clinical Practice Guidelines have progressively refined treatment thresholds in response to emerging evidence [5,29–31]. Consequently, studies conducted in different years or adopting different guidelines naturally applied varying targets for HbA1c, BP, and LDL-C control.

This heterogeneity carries substantial implications for the interpretation of care quality across the literature. First, the lack of a uniform target definition limits the direct comparability between studies, making it difficult to ascertain whether variations in achievement rates reflect actual clinical differences in care delivery or are simply artefacts of the thresholds used. Second, such diversity complicates the longitudinal benchmarking of national care quality, as tracking progress over decades becomes fragmented without a consistent reference point. This ambiguity directly affects the interpretation of findings regarding “stagnation” versus “progress.” For instance, a stable achievement rate over time might mask underlying clinical improvements if targets have become progressively more stringent, while apparent progress might be overstated if less rigorous thresholds are applied. To mitigate this in the current review, we categorised results based on specific thresholds to facilitate a more standardised interpretation of the available evidence. Nevertheless, the lack of a unified reporting framework in the literature obscures the precision of longitudinal assessments and underscores the need for more standardised quality monitoring in future research.

Beyond reflecting temporal and guideline-related variation, this analysis also highlights the limited incorporation of individualised treatment goals. Recent clinical practice guidelines emphasise that HbA1c, BP, and LDL-C targets should be personalised based on a patient’s age, comorbidities, duration of diabetes, and risk of hypoglycaemia [5,32,33]. Yet, this review found only one study for each of the ABC indicators that applied individualised targets [9]. It is important to note that this likely reflects the methodological challenges of operationalising individualised goals in research rather than their absence in clinical practice. In routine care, clinicians generally apply personalised targets according to guideline recommendations, but for research purposes, fixed thresholds are often preferred to enable standardised measurement, statistical comparison, and benchmarking across populations. Nevertheless, this reliance on fixed, universal targets in research limits the ability to capture the real-world complexity of patient-centred care and may not fully reflect the nuances of clinical decision-making.

### Global relevance and broader implications

While this review is focused on Malaysia, its findings offer a valuable case study relevant to other low- and middle-income countries (LMICs) facing a similar, rapidly escalating burden of diabetes. The patterns observed in this review, such as the continuing gap between clinical guidelines and real-world outcomes, which suggests underlying challenges like clinical inertia, inadequate adherence to guidelines, and the strain on PHC systems, are not unique to Malaysia but represent a shared experience across many health systems globally [24,34–36]. The documented trends in HbA1c and BP control, contrasted with modest gains in LDL-C management, provide a microcosm of the differential successes and challenges that can occur within a single system. As such, the lessons learned regarding the importance of robust national registries, the need for balanced policy KPIs, and the critical challenge of translating evidence into practice can inform diabetes care strategies in other nations navigating a similar epidemiological transition.

### Strengths and limitations

The primary strength of this scoping review is its comprehensive and systematic approach. By adhering to the JBI and PRISMA-ScR guidelines and employing a broad search strategy that included both peer-reviewed databases and grey literature, we have provided a comprehensive mapping of the available evidence on this topic for the first time. Our analysis of the research landscape offers valuable insights into its characteristics and trends.

However, this review has several limitations inherent to the scoping review methodology. First, we did not perform a formal quality appraisal or risk of bias assessment of the included studies. The decision to omit a formal study-level quality assessment is consistent with JBI scoping review methodology, which prioritises mapping the range and extent of evidence regardless of quality to provide a broad overview of a research field. While this may limit the ability to grade the strength of individual study findings, the potential impact on the overall interpretation is mitigated by the characteristics of our evidence base. Given that approximately 75% of the large scale publications included in this review rely on the NDR, a national system governed by strict clinical audits and multi-level data validation, the primary data source possesses an inherent degree of methodological rigour and consistency. Consequently, although individual study level bias was not formally assessed, the systemic quality control underlying the majority of the clinical data supports the robustness of our broad interpretations of longitudinal trends.

Second, the conclusions drawn are constrained by the existing evidence base, which may not fully capture the diversity of healthcare contexts across Malaysia. The predominance of studies from urban and better-resourced regions limits the generalisability of findings to rural and underserved areas. However, by systematically including all available studies across multiple states and care settings, this review still provides a comprehensive overview of national trends in T2DM care. While variations in target definitions, data sources, and reporting practices may influence the interpretation of specific outcomes, the overall patterns identified offer valuable insights for policymakers and healthcare planners seeking to improve care quality at the system level.

### Recommendations for policy, practice, and research

The findings of this review have several important implications for improving the quality of T2DM care in Malaysian PHC settings. Achieving meaningful progress in diabetes care requires shifting policy attention from measuring individual indicators toward tackling systemic barriers that hinder care delivery. Policy efforts should prioritise strategies that address workload, consultation time, and resource constraints, while adopting a composite ABC KPI that places equal emphasis on glycaemic, BP, and lipid control. For example, national quality improvement initiatives could incorporate performance indicators such as the proportion of patients achieving simultaneous ABC control and clinic-level benchmarking using registry data, accompanied by regular audit-and-feedback mechanisms to support practice improvement.

In clinical practice, greater focus is needed on overcoming clinical inertia and strengthening adherence to comprehensive ABC management in line with guideline recommendations. Future research should incorporate individualised treatment targets to better reflect real-world care, expand to include rural and underserved populations, and explore the perspectives of key stakeholders to inform more contextually grounded improvements in diabetes management.

## Conclusion

This scoping review provides the first comprehensive synthesis of published evidence on the quality of T2DM care in Malaysian PHC settings, measured through the achievement of ABC control. Despite the growing body of research and policy attention, the findings reveal that glycaemic and BP control continue to show suboptimal levels, while lipid management has shown only modest improvement. The increased use of the NDR as a key data source underscores its pivotal role in supporting large-scale monitoring and research on diabetes care nationwide. However, the findings also highlight continuing gaps between clinical guidelines and real-world outcomes, reflecting enduring systemic, clinical, and patient-level barriers that limit progress in achieving optimal diabetes control.

To advance diabetes care, a more integrated and balanced approach is required at both policy and practice levels. Policymakers should prioritise strategies that strengthen PHC capacity, address clinical inertia, and ensure equitable emphasis on all three ABC indicators in quality improvement initiatives. Continuous investment in and enhancement of the NDR will be vital for maintaining comprehensive, high-quality data to inform evidence-based decisions. Future research should focus on incorporating individualised treatment targets and exploring the perspectives of key stakeholders, which include patients, healthcare providers, and policymakers, to better understand contextual barriers and enablers. Such insights will be instrumental in shaping policies and interventions that foster more effective, patient-centred diabetes management across Malaysia’s PHC system.

## Supporting information

S1 Fig Temporal distribution of publications’ study sites by state/territory (N = 109). (TIF)

S2 Fig Temporal distribution of publications by reported HbA1c targets (N = 80). (TIF)

S3 Fig Temporal distribution of publications by reported BP targets (N = 39). (TIF)

S4 Fig Temporal distribution of publications by reported LDL-C targets (N = 31). (TIF)

S1 Table Search strategies (from inception until December 2024). (DOCX)

S2 Table Screening and retrieval status of reports/ studies with inaccessible full texts. (DOCX)

S3 Table Variables in the standardised data extraction form. (DOCX)

S4 Table Evidence table of the 109 publications included in the scoping review. (DOCX)

S5 Table Summary of funding sources received for the 109 included publications. (DOCX)

S6 Table Funding sources received for the 109 included publications. (DOCX)

S7 Table Preferred Reporting Items for Systematic reviews and Meta-Analyses extension for Scoping Reviews (PRISMA-ScR) Checklist. (DOCX)

## References

[1] Lin X, Xu Y, Pan X, Xu J, Ding Y, Sun X, et al. Global, regional, and national burden and trend of diabetes in 195 countries and territories: an analysis from 1990 to 2025. Sci Rep. 2020;10(1):14790. doi: 10.1038/s41598-020-71908-9 32901098 PMC7478957

[2] International Diabetes Federation. IDF diabetes atlas. 2025.

[3] Ministry of Health Malaysia. National Health and Morbidity Survey (NHMS) 2023: Non-Communicable Diseases and Healthcare Demand: Technical Report. 2024.

[4] Mustapha FI, Aagaard-Hansen J, Lim SC, Nasir NH, Aris T, Bjerre-Christensen U. Variations in the Delivery of Primary Diabetes Care in Malaysia: Lessons to Be Learnt and Potential for Improvement. Health Serv Res Manag Epidemiol. 2020;7. doi: 10.1177/2333392820918744 32313820 PMC7160766

[5] Ministry of Health Malaysia. Clinical Practice Guidelines: Management of Type 2 Diabetes Mellitus (6th Edition). 2020.

[6] American Diabetes Association Professional Practice Committee. 4. Comprehensive Medical Evaluation and Assessment of Comorbidities: Standards of Care in Diabetes-2024. Diabetes Care. 2024;47(Suppl 1):S52–76. doi: 10.2337/dc24-S004 38078591 PMC10725809

[7] Ministry of Health Malaysia. National Diabetes Registry Report 2023. 2024.

[8] Mafauzy M, Hussein Z, Chan SP. The status of diabetes control in Malaysia: results of DiabCare 2008. Med J Malaysia. 2011;66(3):175–81. 22111435

[9] Wan KS, Moy FM, Mustapha FI, Ismail M, Hairi NN. Changes in body mass index, glycosylated hemoglobin A1C, blood pressure, and LDL-cholesterol among type 2 diabetes patients in Malaysia: A population-based longitudinal study. J Diabetes. 2021;13(11):915–29. doi: 10.1111/1753-0407.13206 34142456

[10] Peters MDJ, Marnie C, Tricco AC, Pollock D, Munn Z, Alexander L, et al. Updated methodological guidance for the conduct of scoping reviews. JBI Evid Synth. 2020;18(10):2119–26. doi: 10.11124/JBIES-20-00167 33038124

[11] Tricco AC, Lillie E, Zarin W, O’Brien KK, Colquhoun H, Levac D, et al. PRISMA Extension for Scoping Reviews (PRISMA-ScR): Checklist and Explanation. Ann Intern Med. 2018;169(7):467–73. doi: 10.7326/M18-0850 30178033

[12] Ministry of Health Malaysia. National Strategic Plan for Non-Communicable Disease (NSP-NCD) 2016-2025. Ministry of Health Malaysia. 2016. https://www.moh.gov.my/moh/resources/Penerbitan/Rujukan/NCD/National%20Strategic%20Plan/FINAL_NSPNCD.pdf

[13] Hussein Z, Taher SW, Gilcharan Singh HK, Chee Siew Swee W. Diabetes care in Malaysia: problems, new models, and solutions. Ann Glob Health. 2015;81(6):851–62. 27108152 10.1016/j.aogh.2015.12.016

[14] Ab Hamid J, Juni MH, Abdul Manaf R, Syed Ismail SN, Lim PY. Spatial Accessibility of Primary Care in the Dual Public-Private Health System in Rural Areas, Malaysia. Int J Environ Res Public Health. 2023;20(4):3147. doi: 10.3390/ijerph20043147 36833838 PMC9959538

[15] Lim HM, Sivasampu S, Khoo EM, Mohamad Noh K. Chasm in primary care provision in a universal health system: Findings from a nationally representative survey of health facilities in Malaysia. PLoS One. 2017;12(2):e0172229. doi: 10.1371/journal.pone.0172229 28196113 PMC5308860

[16] Ministry of Health Malaysia. Manual pengguna audit klinikal diabetes di fasiliti kesihatan. 2008.

[17] Feisul MI. National Diabetes Registry Report, Volume 1, 2009-2012. Kuala Lumpur. 2013.

[18] Wan KS, Hairi NN, Mustapha F, Ismail M, Mohd Yusoff MF, Moy FM. Five-year LDL-cholesterol trend and its predictors among type 2 diabetes patients in an upper-middle-income country: a retrospective open cohort study. PeerJ. 2022;10:e13816. doi: 10.7717/peerj.13816 36317122 PMC9617547

[19] Abas MZ, Hairi NN, Choo WY, Wan KS, Li K. Unravelling the association of glycosylated haemoglobin A1c, blood pressure, and LDL-cholesterol (ABC) with all-cause mortality in Type 2 diabetes patients: insights from a middle-income country. J Diabetes Metab Disord. 2025;24(1):111. doi: 10.1007/s40200-025-01620-w 40321425 PMC12043549

[20] Ministry of Health Malaysia. SIQ investigation quality of diabetes care at MOH healthcare facilities: glycaemic control. 2008.

[21] de Boer IH, Bangalore S, Benetos A, Davis AM, Michos ED, Muntner P, et al. Diabetes and Hypertension: A Position Statement by the American Diabetes Association. Diabetes Care. 2017;40(9):1273–84. doi: 10.2337/dci17-0026 28830958

[22] Mourad J-J, Le Jeune S. Blood pressure control, risk factors and cardiovascular prognosis in patients with diabetes: 30 years of progress. J Hypertens Suppl. 2008;26(3):S7–13. doi: 10.1097/01.hjh.0000334072.97080.33 19363847

[23] Pan C-W, Wang S, Xu C-L, Song E. Combined effect of glycemic and blood pressure control on diabetic retinopathy among Chinese with type-2 diabetes mellitus. Diabetol Metab Syndr. 2018;10:73. doi: 10.1186/s13098-018-0377-7 30302129 PMC6167778

[24] Wan KS, Moy FM, Mohd Yusof K, Mustapha FI, Mohd Ali Z, Hairi NN. Clinical inertia in type 2 diabetes management in a middle-income country: A retrospective cohort study. PLoS One. 2020;15(10):e0240531. doi: 10.1371/journal.pone.0240531 33035261 PMC7546487

[25] Pourhabibi N, Mohebbi B, Sadeghi R, Shakibazadeh E, Sanjari M, Tol A, et al. Determinants of Poor Treatment Adherence among Patients with Type 2 Diabetes and Limited Health Literacy: A Scoping Review. J Diabetes Res. 2022;2022:2980250. doi: 10.1155/2022/2980250 35832786 PMC9273343

[26] Chauke GD, Nakwafila O, Chibi B, Sartorius B, Mashamba-Thompson T. Factors influencing poor medication adherence amongst patients with chronic disease in low-and-middle-income countries: A systematic scoping review. Heliyon. 2022;8(6):e09716. doi: 10.1016/j.heliyon.2022.e09716 35770147 PMC9234585

[27] Jin J, Sklar GE, Min Sen Oh V, Chuen Li S. Factors affecting therapeutic compliance: A review from the patient’s perspective. Ther Clin Risk Manag. 2008;4(1):269–86. doi: 10.2147/tcrm.s1458 18728716 PMC2503662

[28] Elnaem MH, Mohamed MHN, Huri HZ, Shah ASM. Effectiveness and prescription pattern of lipid-lowering therapy and its associated factors among patients with type 2 diabetes mellitus in Malaysian primary care settings. Ther Clin Risk Manag. 2019;15:137–45. doi: 10.2147/TCRM.S182716 30705590 PMC6342220

[29] Ministry of Health Malaysia. Clinical Practice Guidelines: Management of Type 2 Diabetes Mellitus. 2015.

[30] Ministry of Health Malaysia. Clinical Practice Guidelines: Management of Type 2 Diabetes Mellitus. 2009.

[31] Ministry of Health Malaysia. Clinical practice guidelines: Management of type 2 diabetes mellitus (3rd edition). Ministry of Health Malaysia. 2004.

[32] American Diabetes Association. Standard of Care in Diabetes - 2023. The Journal of Clinical and Applied Research and Education. 2022;46.

[33] National Institute for Health and Care Excellence. Type 2 Diabetes in Adults: Management. 2015. 26741015

[34] Almigbal TH, Alzarah SA, Aljanoubi FA, Alhafez NA, Aldawsari MR, Alghadeer ZY, et al. Clinical Inertia in the Management of Type 2 Diabetes Mellitus: A Systematic Review. Medicina (Kaunas). 2023;59(1):182. doi: 10.3390/medicina59010182 36676805 PMC9866102

[35] Teh XR, Lim MT, Tong SF, Husin M, Khamis N, Sivasampu S. Quality of hypertension management in public primary care clinics in Malaysia: An update. PLoS One. 2020;15(8):e0237083. doi: 10.1371/journal.pone.0237083 32780769 PMC7418969

[36] Wan KS, Moy FM, Mohd Yusoff MF, Mustapha F, Ismail M, Mat Rifin H, et al. Treatment intensification and therapeutic inertia of antihypertensive therapy among patients with type 2 diabetes and hypertension with uncontrolled blood pressure. Sci Rep. 2024;14(1):12625. doi: 10.1038/s41598-024-63617-4 38824234 PMC11144228

