# Supplementary material for: The quality of care for type 2 diabetes mellitus management in Malaysian primary health care settings: A scoping review of ABC (glycated haemoglobin A1c, blood pressure, and LDL-cholesterol)
Source: PLoS One. 2026 Jul 31;21(7):e0355227. doi: 10.1371/journal.pone.0355227 (PMC13426932; doi:10.1371/journal.pone.0355227)
Supplement: S2 Table — (DOCX) [file pone.0355227.s006.docx]

**S2 Table. Screening and retrieval status of reports/ studies with inaccessible full texts**

| **No.** | **Source of report** | **Reports/ studies** | **Title/ abstract screen** | **Remarks** | **Attempted to contact corresponding author at least twice via email/ Researchgate** |
| --- | --- | --- | --- | --- | --- |
| 1 | Identified via databases (Results of search terms from the four key databases) | Kong, J. P., Ayub, A. B., & Bau, R. A. (2016). TLC vs Simvastatin vs Simvastatin and TLC in hypercholesterolemia: a 3-months controlled trial in a Malaysia primary health care clinic. Nutrition & Food Science, 46(3), 344-362. | Not relevant | Study population was not DM patients | Yes |
| 2 | Identified via databases (Results of search terms from the four key databases) | Gillani, S. W., Syed Sulaiman, S. A., Abdul, M. I., & Saad, S. Y. (2018). Physical disability and diabetes mellitus; qualitative exploration of patients' perception and behavior. Current Diabetes Reviews, 14(5), 472-480. | Not relevant | Not generalisable due to qualitative study nature | Yes |
| 3 | Identified via databases (Results of search terms from the four key databases) | Kassab, Y. W., Aldahoul, H. K., Humayra, S., Saad, E. M. S., Iqbal, M. Z., Naqvi, A. A., ... & Iqbal, M. S. (2020). Prevalence of UTI and its association with glycemic levels, common uropathogens involved, and antibiotic resistance among diabetic patients. Latin American Journal of Pharmacy, 39(9), 1840-1847. | Not relevant | Not primary care | Yes |
| 4 | Identified via databases (Results of search terms from the four key databases) | Latif, H. A., Hamid, M. R. A., Azizan, N. A., & Jemain, A. A. (2017, May). Path modelling of antecedent of diabetes mellitus on blood glucose measurements. In AIP Conference Proceedings (Vol. 1842, No. 1, p. 020008). AIP Publishing LLC. | Not relevant | Purely statistical modelling. Not measuring ABC parameter of diabetes patients. | Yes |
| 5 | Identified via databases (Results of search terms from the four key databases) | Dziehan, M., Cheat, W. Y., Thiruvanackan, K. A., Azmiah, N., Hui, C. B., Sariam, N., ... & Ali, S. M. (2009, August). A prospective multicentre study of pharmacist initiated programme of'Medication Therapy Management'(MTM) in Government Primary Health Clinic in the state of Selangor. In PHARMACY WORLD & SCIENCE (Vol. 31, No. 4, pp. 498-498). VAN GODEWIJCKSTRAAT 30, 3311 GZ DORDRECHT, NETHERLANDS: SPRINGER. | Not relevant | Not generalisable as the study population include on uncontrolled DM patients | Yes |
| 6 | Identified via databases (Results of search terms from the four key databases) | K., Makmon, and Ahmad Zaid Fattah Azman. IDF23-0163Self-efficacy and Self-stigma Its Association with Diabetes Self-care in Selangor, Malaysia: A Cross-sectional Study. Elsevier, 2024. | Not relevant | Questionnaire-based only. No lab results. | Yes |
| 7 | Identified via other methods (Backward citation tracking of the reference list/ known grey literature) | Johari, R. (2006). A study on the adequacy of outpatient management of type II diabetes mellitus cases in MOH hospitals and health centres. Institute of Health Management, Ministry of Health Malaysia. | Cannot be decided. Need full text. |  | No. Unable to trace corresponding author. |
| 8 | Identified via other methods (Backward citation tracking of the reference list/ known grey literature) | Rahman, N. A. A., Ismail, A. A. S., & Yaacob, N. A. (2008). Use of CPG on Management of Type 2 Diabetes among Diabetes Care Teams in North-East Malaysia. International Medical Journal, 15(3). | Not relevant | This study was among healthcare provider, not DM patients | Yes |
| 9 | Identified via other methods (Backward citation tracking of the reference list/ known grey literature) | Fauziah MN, Suhaiza S. (2004). A review of diabetic complicatinos amongst poorly controlled diabetes at 8 health clinics in Kelantan. NCD Malaysia, 3, 13-16. | Not relevant | Not generalisable as the study population included only uncontrolled DM patients | No. Unable to trace corresponding author. |
| 10 | Identified via other methods (Backward citation tracking of the reference list/ known grey literature) | Norimah, A. K., & Abu Bakar, A. A. H. (1993). Food intake and anthropometric status of diabetics attending Universiti Kebangsaan Malaysia (UKM) outpatient clinic. Proc Nutr Soc Mat, 8, 16-22. | Cannot be decided. Need full text. |  | Yes |
| 11 | Identified via other methods (Backward citation tracking of the reference list/ known grey literature) | Ruzita AT (1998). Keberkesanan kaunseling pemakanan berkelompok ke atas pengawalan diabetes di kalangan individu yang mengalami diabetes melitus jenis II di kawasan FELDA, semibandar dan bandar di Malaysia. Tesis Dr. Fal. Universiti Kebangsaan Malaysia. | Cannot be decided. Need full text. |  | No. Unable to trace corresponding author. |
| 12 | Identified via other methods (Backward citation tracking of the reference list/ known grey literature) | Ambigapathy, R., Ambigapathy, S., & Ling, H. M. (2003). A knowledge, attitude and practice (KAP) study of diabetes mellitus among patients attending Klinik Kesihatan Seri Manjung. NCD Malaysia, 2(2), 6-16. | Cannot be decided. Need full text. |  | No. Unable to trace corresponding author. |
| 13 | Identified via other methods (Backward citation tracking of the reference list/ known grey literature) | Ding HJ, Chan SC. Comparing morbidity patterns in two government health centers and four private GP clinics in the Kinta district. Family Physician. 2003;12(2&3):25-29 | Not relevant | Comparing pattern of diseases like URTI, emergency cases, chrinic diseases etc. seen in the facilities. Not study on DM patients. | No. Unable to trace corresponding author. |
| 14 | Identified via other methods (Backward citation tracking of the reference list/ known grey literature) | Pillay, R. P., & Hin, L. E. (1960). Incidence of diabetes mellitus in Malaya. The Medical journal of Malaya, 14, 242-244. | Cannot be decided. Need full text. |  | No. Unable to trace corresponding author. |
| 15 | Identified via other methods (Backward citation tracking of the reference list/ known grey literature) | Suhaiza, S., Ahmad Nasir, M., Jeriah, I., Abdul Aziz Al-Safi, I., Wan Mohamad, W. B., & Mafauzy, M. (2004). Glycaemic control among type 2 diabetic patients in Kelantan. NCD Malaysia, 3, 2-5. | Cannot be decided. Need full text. |  | No. Unable to trace corresponding author. |
| 16 | Identified via other methods (Backward citation tracking of the reference list/ known grey literature) | Institute for Health Management (2006) A study on the adequacy of Outpatient Management of Essential Hypertension in MOH Hospitals and Health Centres | Not relevant | Study population was not DM patients | No. Unable to trace corresponding author. |
| 17 | Identified via other methods (Backward citation tracking of the reference list/ known grey literature) | Ayadurai, S., Sunderland, V. B., Tee, L. B., & Hattingh, H. L. (2018). Consensus validation of Simpler™: A tool to improve pharmacist delivery of quality, evidence-based diabetes care. Current Diabetes Reviews, 14(6), 565-575. | Not relevant | This study was among healthcare provider, not DM patients | Yes |
| 18 | Identified via other methods (Backward citation tracking of the reference list/ known grey literature) | NHMS 2006. Diabetes mellitus among adults 30 years and above. Public Health Institute, Ministry of Health Malaysia; 2007. | Not relevant | This study was conducted among general population, not DM among DM patients | Yes |

As mentioned in the main manuscript, this review was conducted as a focused component of a broader scoping review project (MyABCMap), which aimed to map the quality of care for type 2 diabetes mellitus (T2DM), hypertension, and dyslipidaemia in Malaysia. During the initial screening phase of the parent MyABCMap review, 18 records were excluded because their full texts could not be retrieved.

To ensure the comprehensiveness of this specific review on T2DM ABC control, we reassessed all 18 previously excluded records. Based on a screening of titles and abstracts, 12 of these reports were found to be irrelevant to the current study’s objectives. The eligibility of the remaining six reports could not be definitively determined without full-text access. However, these six records are historical (most were published than 20 years ago), and we believe their exclusion has a minimal impact on the overall findings compared to the 109 studies included in this synthesis.

Regarding retrievability efforts, 10 reports where contact information was available, we attempted to contact the corresponding authors via email or professional networks (e.g., ResearchGate) at least twice, with attempts spaced a minimum of two weeks apart. For the remaining eight reports, no valid contact channels could be traced.
