# Supplementary material for: The quality of care for type 2 diabetes mellitus management in Malaysian primary health care settings: A scoping review of ABC (glycated haemoglobin A1c, blood pressure, and LDL-cholesterol)
Source: PLoS One. 2026 Jul 31;21(7):e0355227. doi: 10.1371/journal.pone.0355227 (PMC13426932; doi:10.1371/journal.pone.0355227)
Supplement: S3 Table — (DOCX) [file pone.0355227.s007.docx]

**S3 Table. Variables in the standardised data extraction form**

This table outlines the components and variables considered in the standardised data extraction form.

**S3(a) Table. Variables used in the final data extraction form**

Variables marked as "Added/Refined" indicate changes made following the pilot test of 10 studies to ensure the form captured the nuances of Malaysian primary care data. A section for "Removed Variables" is provided at the end to justify the exclusion of certain data points from the final extraction phase.

| **Domain** | **Variable Name** | **Description / Options** | **Status after Pilot Test** |
| --- | --- | --- | --- |
| **Study Characteristics** | No. | Unique identifier for each study. | Original |
|  | Author & Year | Primary author and year of publication. | Original |
|  | Study Location | State or territory in Malaysia. | **Refined:** Added specific state dropdowns to map geographical distribution. |
| **Methodology** | Study Design | e.g., Cross-sectional, Cohort, Registry-based audit. | Original |
|  | Sample Size | Total number of T2DM patients included. | Original |
|  | Data Source | e.g., NDR, ADCM, EMR, Manual Medical Records.  Note: Only extracted for publications with N ≥ 1000. | **Added:** To identify the origin of large-scale data |
|  | Year of Data Collection | The actual year(s) the clinical data was recorded. | **Added:** Critical to distinguish from publication year for accurate trend mapping. |
| **HbA1c Outcomes** | Targets Reported | Specific thresholds used (e.g., <6.5%, <7.0%, ≤8.0%). | **Refined:** Expanded to allow multiple entries due to threshold heterogeneity. |
|  | Achievement Rate (%) | Percentage of patients meeting the specific target. | Original |
|  | Mean / Median HbA1c | Central tendency value for the study population. | Original |
| **Blood Pressure** | BP Targets Reported | e.g., <130/80, ≤135/75, <140/90 mmHg. | **Refined:** Expanded to capture combined vs. single (SBP/DBP) targets. |
|  | Achievement Rate (%) | Percentage meeting combined or single targets. | Original |
|  | Mean SBP / DBP | Mean or median values in mmHg. | Original |
| **LDL-Cholesterol** | LDL-C Targets Reported | e.g., <2.6 mmol/L, ≤2.6 mmol/L. | **Refined:** Added options for newer, stricter targets (e.g., <1.8 or <1.4). |
|  | Achievement Rate (%) | Percentage meeting the lipid target. | Original |
|  | Mean / Median LDL-C | Value reported in mmol/L. | Original |

**S3(b) Table. Variables Removed Following Pilot Test**

**Note:** The variables listed below were removed only from the formal data extraction phase to enhance efficiency and maintain focus on information crucial to answering the primary study objectives. However, all such information was fully considered during the title, abstract, and full-text screening phases to ensure that each study strictly fulfilled the predefined eligibility criteria for this scoping review.

| **Removed Variable** | **Justification for Removal** |
| --- | --- |
| Study Objectives / Primary Aims | The pilot test confirmed that primary studies had diverse objectives (e.g., tool validation, specific interventions). These were removed from extraction to focus solely on clinical ABC outcomes. |
| Detailed Ethnicity Breakdown | While reported in some studies, inconsistent reporting across all 109 publications made it unfeasible for aggregate mapping without significant data gaps. |
| Duration of Diabetes | Found to be poorly or inconsistently reported in older and smaller studies; deemed a secondary factor that did not directly answer the primary question of ABC achievement rates. |
| Reference or Guideline Source Used for ABC Thresholds | It was observed that most studies reported the numerical ABC thresholds (e.g., HbA1c <7%, BP <130/80 mmHg) but did not explicitly state the guideline or reference used to define these thresholds. Due to this inconsistent reporting across studies, extracting the specific reference source was not feasible for systematic mapping. |
| Specific Medication Names/Dosages | Pilot revealed that focusing on drug-specific data was better suited for a systematic review of interventions rather than a scoping review mapping overall quality of care. |
| List of All Comorbidities | Excluded as our objective focused on the general T2DM population rather than sub-populations with specific complications (e.g., CKD or retinopathy). |
