# Supplementary material for: The quality of care for type 2 diabetes mellitus management in Malaysian primary health care settings: A scoping review of ABC (glycated haemoglobin A1c, blood pressure, and LDL-cholesterol)
Source: PLoS One. 2026 Jul 31;21(7):e0355227. doi: 10.1371/journal.pone.0355227 (PMC13426932; doi:10.1371/journal.pone.0355227)
Supplement: S4 Table — (DOCX) [file pone.0355227.s008.docx]

**S4 Table. Evidence table of the 109 publications included in the scoping review**

| **No.** | **Author (Year Published)** | **Characteristic of Study** | **HbA1c** | **Blood Pressure** | **LDL-C** |
| --- | --- | --- | --- | --- | --- |
|  | (Ong et al., 2022) | Study design: Cross-Sectional  Sample size: 311  Study location: Pulau Pinang  Year data collected: 2019 | HbA1c <7: 33.4%  HbA1c 7.0- 8.5: 32.5%  HbA1c > 8.6: 31.2%  Median HbA1c (%): 7.5 | n/a | n/a |
|  | (Gunggu et al., 2016) | Study design: Cross-Sectional  Sample size: 400  Study location: Sarawak  Year data collected: 2013 | HbA1c <6.5: 59.1%  HbA1c ≥ 6.5: 40.9%  Mean HbA1c (%): 6.67 | n/a | n/a |
|  | (Lee et al., 2020) | Study design: Cross-Sectional  Sample size: 11,736  Study location: Selangor, Johor  Year data collected: 2017 | HbA1c ≤ 7: 34.4%  HbA1c ≤ 8: 53%  Mean HbA1c (%): 8.3 | BP ≤ 135/75: 26.2%  BP ≤ 130/80: 28.8%  BP ≤ 140/80: 44.6%  Mean SBP (mmHg): 137.8  Mean DBP (mmHg): 77.8 | LDL-C ≤2.6 mmol/L: 39%  LDL-C > 2.6 mmol/L: 61%  Mean LDL-C (mmol/L): 3 |
|  | (Naserrudin et al., 2022) | Study design: Cross-Sectional  Sample size: 22,345  Study location: Sabah  Year data collected: 2011 | HbA1c <6.5: 46.6%  HbA1c ≥ 6.5: 53.4% | n/a | n/a |
|  | (Husain et al., 2023) | Study design: Cross-Sectional  Sample size: 703  Study location: Terengganu  Year data collected: 2018 | HbA1c <6.5: 24.5%  HbA1c ≥ 6.5: 75.5%  Mean HbA1c (%): 8.41 | n/a | n/a |
|  | (Jamaluddin & Mohamed Kamel, 2024) | Study design: Cross-Sectional  Sample size: 4028  Study location: Perak  Year data collected: 2020 | Prevalence of HbA1c:  HbA1c <7: 36.4%  HbA1c ≥7: 63.6%  Median HbA1c (%): 7.7 | Blood Pressure Target:  SBP <140: 56.8%  SBP ≥ 140: 43.2%  DBP <80: 64.4%  DBP ≥ 80: 35.6%  Median SBP (mmHg): 137  Median DBP (mmHg): 75 | LDL-C <2.6(mmol/L): 47.5%  LDL-C ≥ 2.6(mmol/L): 52.5%  Median LDL-C (mmol/L): 2.6 |
|  | (Ab Rahman et al., 2022) | Study design: Cohort  Sample size: 2696  Study location: Selangor, Johor  Year data collected: 2018 | HbA1c ≤ 7: 34.5%  HbA1c > 7: 65.5%  Mean HbA1c (%): 8.3  Median HbA1c (%): 7.8 | n/a | n/a |
|  | (Nordin et al., 2021) | Study design: Cross-Sectional  Sample size: 772  Study location: Kelantan  Year data collected: 2019 | HbA1c ≤6.5: 16.3%  HbA1c > 6.5: 83.7%  Mean HbA1c (%): 8.91 | n/a | n/a |
|  | (Lim et al., 2019) | Study design: Cross-Sectional  Sample size: 2960  Study location: Selangor, Johor  Year data collected: 2016 | Mean HbA1c (%): 8.4 | Mean SBP (mmHg): 137.7  Mean DBP (mmHg): 77.7 | Mean LDL-C (mmol/L): 3 |
|  | (Nasir et al., 2022) | Study design: Cross-Sectional  Sample size: 350  Study location: Kelantan  Year data collected: 2020 | HbA1c <7: 73.7%  HbA1c ≥7: 26.3%  Median HbA1c (%): 8.2 | n/a | n/a |
|  | (Chew et al., 2021) | Study design: Cohort  Sample size: 700  Study location: Selangor  Year data collected: 2015 | Mean HbA1c (%): 8.0 | Mean SBP (mmHg): 137  Mean DBP (mmHg): 79 | Mean LDL-C (mmol/L): 3 |
|  | (Syed Soffian et al., 2019) | Study design: Cross-Sectional  Sample size: 23,557  Study location: Kedah  Year data collected: 2017 | HbA1c<6.5: 15.6%  HbA1c 6.5- 7.4: 15.2%  HbA1c 7.5- 8.4: 11.2%  HbA1c 8.5- 10.0: 13.5%  HbA1c >10.0: 15.8%  Mean HbA1c (%): 8.4 | n/a | n/a |
|  | (Wan et al., 2022) | Study design: Cohort  Sample size: 18,312  Study location: Negeri Sembilan  Year data collected: 2013 | n/a | n/a | 2013 Mean LDL-C (mmol/L): 2.95  2014 Mean LDL-C (mmol/L): 2.92  2015 Mean LDL-C (mmol/L): 2.83  2016 Mean LDL-C (mmol/L): 2.8  2017 Mean LDL-C (mmol/L): 2.76 |
|  | (Chamhuri et al., 2022) | Study design: Cross-Sectional  Sample size: 200  Study location: Kuala Lumpur  Year data collected: 2021 | HbA1c <7: 33.0%  HbA1c ≥7: 67.0%  Mean HbA1c (%): 8.0 | Mean SBP (mmHg): 138.2  Mean DBP (mmHg): 80.1 | n/a |
|  | (Lim et al., 2021) | Study design: Cross-Sectional  Sample size: 1461  Study location: Selangor, Johor  Year data collected: 2019 | Mean HbA1c (%): 7.7  Median HbA1c (%): 7.2 | n/a | n/a |
|  | (Lai et al., 2020) | Study design: Validation study  Sample size: 100  Study location: Kuala Lumpur  Year data collected: 2016 | HbA1c <7: 30.1%  HbA1c ≥7: 69.8% | n/a | n/a |
|  | (Abdullah et al., 2020) | Study design: Cross-Sectional  Sample size: 427  Study location: Perak  Year data collected: 2018 | HbA1c ≤6.5: 31.1% | BP ≤ 135/75 mmHg: 69.6% | n/a |
|  | (Sazlina et al., 2020) | Study design: Cross-Sectional  Sample size: 506  Study location: Selangor  Year data collected: 2018 | HbA1c ≤ 7: 38.9%  HbA1c 7.1- 8.0: 19.2%  HbA1c > 8.0: 41.9% | Mean SBP (mmHg): 138.7  Mean DBP (mmHg): 73.3 | Median LDL-C (mmol/L): 2.7 |
|  | (Dhillon et al., 2019) | Study design: Cross-Sectional  Sample size: 150  Study location: Kuala Lumpur  Year data collected: 2017 | HbA1c ≤6.5: 21%  HbA1c >6.5: 79%  Mean HbA1c (%): 8 | Mean SBP (mmHg): 139  Mean DBP (mmHg): 76 | n/a |
|  | (Leelavathi et al., 2013) | Study design: Cross-Sectional  Sample size: 151  Study location: Kuala Lumpur  Year data collected: 2011 | HbA1c <6.5: 21%  HbA1c ≥ 6.5: 79% | n/a | n/a |
|  | (Zhu et al., 2019) | Study design: Cross-Sectional  Sample size: 322  Study location: Putrajaya  Year data collected: 2019 | Median HbA1c (%): 7.4 | Mean SBP (mmHg): 133.2  Mean DBP (mmHg): 83.7 | Median LDL-C (mmol/L): 3 |
|  | (Tharek et al., 2018) | Study design: Cross-Sectional  Sample size: 340  Study location: Selangor  Year data collected: 2015 | HbA1c <6.5: 13.5%  HbA1c ≥ 6.5: 86.5%  Mean HbA1c (%): 7.99 | BP ≥ 140/80 mmHg: 60.3%  BP < 140/80 mmHg: 39.7% | n/a |
|  | (Goh et al., 2020) | Study design: Validation study  Sample size: 120  Study location: Kuala Lumpur  Year data collected: 2019 | HbA1c ≤ 7: 40.7%  HbA1c > 7: 59.2%  Median HbA1c (%): 7.5 | n/a | n/a |
|  | (Chan, 2005) | Study design: Cross-Sectional  Sample size: 350  Study location: Melaka  Year data collected: 2003 | n/a | BP < 130/80mmHg: 3.1%  Mean SBP (mmHg): 143.2  Mean DBP (mmHg): 85.1 | n/a |
|  | (Mahmood et al., 2016) | Study design: Cross-Sectional  Sample size: 706  Study location: Johor  Year data collected: 2013 | HbA1c ≤6.5: 32%  HbA1c >6.5: 68%  Mean HbA1c (%): 7.8 | BP < 130/80mmHg: 68.3%  BP ≥130/80mmHg: 31.7% | n/a |
|  | (Kaur et al., 2013) | Study design: Cross-Sectional  Sample size: 2508  Study location: Kuala Lumpur, Putrajaya, Selangor  Year data collected: 2009 | HbA1c ≤ 8.5: 61.3%  HbA1c > 8.5: 38.7%  Mean HbA1c (%): 8.36 | n/a | n/a |
|  | (Ching et al., 2013) | Study design: Cross-Sectional  Sample size: 240  Study location: Selangor  Year data collected: 2011 | Mean HbA1c (%): 8.7 | Mean SBP (mmHg): 138  Mean DBP (mmHg): 82 | n/a |
|  | (B.-H. Chew et al., 2015) | Study design: Cross-Sectional  Sample size: 700  Study location: Selangor  Year data collected: 2012 | HbA1c <7, 26.4%  HbA1c <6.5, 16.7% | BP > 130/80mmHg: 69.5%  BP ≤ 130/80mmHg: 30.5% | LDL-C ≤2.6(mmol/L): 40.1%  LDL-C > 2.6(mmol/L): 59.9% |
|  | (Wong & Rahimah, 2004) | Study design: Cross-Sectional  Sample size: 1031  Study location: Sarawak  Year data collected: 2003 | HbA1c <6.5: 28.0%  HbA1c 6.5- 7.5: 34%  HbA1c <7: 62%  HbA1c > 7.5: 38%  HbA1c <8: 73%  Mean HbA1c (%): 7.4 | n/a | n/a |
|  | (Chen et al., 2022) | Study design: Cross-Sectional  Sample size: 295  Study location: Johor  Year data collected: 2019 | Mean HbA1c (%): 7.5 | n/a | n/a |
|  | (Tan & Ng, 2023) | Study design: Cross-Sectional  Sample size: 418  Study location: Kuala Lumpur  Year data collected: 2019 | HbA1c ≤6.5: 24.3%  HbA1c 6.6- 8.0: 35.5%  HbA1c ≥ 8.1: 40.2%  Mean HbA1c (%): 7.98 | n/a | n/a |
|  | (Chin et al., 2023) | Study design: Cross-Sectional  Sample size: 386  Study location: Johor  Year data collected: 2018 | Mean HbA1c (%): 8.3 | n/a | n/a |
|  | (Yahya et al., 2023a) | Study design: Cross-Sectional  Sample size: 300  Study location: Selangor  Year data collected: 2022 | Mean HbA1c (%): 8.38 | n/a | n/a |
|  | (Yahya et al., 2023b) | Study design: Cross-Sectional  Sample size: 300  Study location: Selangor  Year data collected: 2020 | HbA1c <7%: 2.3%  HbA1c 7.0- 8.5: 14.3%  HbA1c > 8.5%: 83.3%  Mean HbA1c (%): 9.65 | n/a | n/a |
|  | (Samat et al., 2024) | Study design: Cross-Sectional  Sample size: 144  Study location: Selangor  Year data collected: 2018 | Mean HbA1c (%): 8.471 | n/a | n/a |
|  | (Bujang et al., 2021) | Study design: Cross-Sectional  Sample size: 536  Study location: Negeri Sembilan  Year data collected: 2015 | HbA1c <7: 32.6%  HbA1c ≥7: 67.4% | n/a | n/a |
|  | (Wan et al., 2021a) | Study design: Cross-Sectional  Sample size: 17592  Study location: Negeri Sembilan  Year data collected: 2015 | HbA1c <7%: 42.0%  Mean HbA1c (%): 7.87 | BP < 130/80mmHg: 22.3%  Mean SBP (mmHg): 134.8  Mean DBP (mmHg): 78.3 | LDL-C < 2.6(mmol/L): 38.1%  Mean LDL-C (mmol/L): 2.91 |
|  | (Lee et al., 2022) | Study design: Cross-Sectional  Sample size: 425  Study location: Selangor  Year data collected: 2017 | HbA1c <6.5: 23.3%  HbA1c ≥6.5: 76.7% | BP < 140/80mmHg: 43.5%  BP ≥ 140/80mmHg: 56.5% | n/a |
|  | (Nordin et al., 2020) | Study design: Cross-Sectional  Sample size: 772  Study location: Kelantan  Year data collected: 2019 | HbA1c ≤6.5: 16.3%  HbA1c > 6.5: 83.7% | n/a | n/a |
|  | (Rashid et al., 2020) | Study design: Cross-Sectional  Sample size: 329  Study location: Selangor  Year data collected: 2013 | Mean HbA1c (%): 8.0 | n/a | n/a |
|  | (Lee et al., 2019) | Study design: Cross-Sectional  Sample size: 196  Study location: Negeri Sembilan  Year data collected: 2016 | Median HbA1c (%): 8.1 | n/a | n/a |
|  | (Rasid et al., 2020) | Study design: Cross-Sectional  Sample size: 271  Study location: Kuala Lumpur  Year data collected: 2019 | HbA1c ≤6.5: 22.1%  HbA1c > 6.5: 77.9%  Median HbA1c (%): 7.7 | n/a | Median LDL-C (mmol/L): 2.4 |
|  | (Tan & Ismail, 2020) | Study design: Cross-Sectional  Sample size: 289  Study location:  Selangor  Year data collected: 2018 | HbA1c ≤6.5: 22.7%  HbA1c > 6.5: 77.3%  Mean HbA1c (%): 8.26 | n/a | n/a |
|  | (Papo et al., 2019) | Study design: Cross-Sectional  Sample size: 331  Study location: Sabah  Year data collected: 2017 | HbA1c ≤6.5: 56.2%  HbA1c > 7: 43.8%  Median HbA1c (%): 7 | n/a | n/a |
|  | (Moy Foong & Yew Sheng, 2019) | Study design: Cohort  Sample size: 377  Study location: Selangor  Year data collected: 2011 | n/a | Mean SBP (mmHg): 141.77 | n/a |
|  | (Abdullah et al., 2017) | Study design: Cross-Sectional  Sample size: 154  Study location: Pahang  Year data collected: 2014 | n/a | BP ≤ 130/80mmHg: 33.1%  BP >130/80mmHg: 51.9%  BP >130/≤80mmHg: 10.4%  BP ≤130/>80mmHg: 4.5% | n/a |
|  | (Jusoh et al., 2018) | Study design: Cross-Sectional  Sample size: 180  Study location: Not specified  Year data collected: 2012 | HbA1c ≤ 7: 17.8%  HbA1c > 7: 82.2%  Median HbA1c (%): 8.7 | n/a | n/a |
|  | (Chin et al., 2017) | Study design: Validation study  Sample size: 114  Study location: Kuala Lumpur  Year data collected: 2014 | HbA1c ≤ 7: 47.2%  HbA1c > 7: 52.8%  Median HbA1c (%): 7.1 | n/a | n/a |
|  | (Mohd Aznan et al., 2018) | Study design: Cross-Sectional  Sample size: 400  Study location: Pahang  Year data collected: 2010 | Mean HbA1c (%): 8.3 | BP ≤ 130/80mmHg: 54%  BP >130/80mmHg: 49% | n/a |
|  | (Chew et al., 2017) | Study design: Cohort  Sample size: 336  Study location: Selangor  Year data collected: 2016 | Mean HbA1c (%): 8.4 | BP < 130/80mmHg: 33% | LDL-C ≤ 2.6(mmol/L): 41.2% |
|  | (Hashim et al., 2016) | Study design: Cross-Sectional  Sample size: 204  Study location: Kuala Lumpur  Year data collected: 2011 | HbA1c<6.5: 16.2%  HbA1c > 6.5: 83.3% | n/a | n/a |
|  | (W. L. Tan et al., 2015) | Study design: Cross-Sectional  Sample size: 461  Study location: Kedah  Year data collected: 2012 | Mean HbA1c (%): 8.79 | n/a | n/a |
|  | (Azura et al., 2012) | Study design: Cross-Sectional  Sample size: 254  Study location: Kelantan  Year data collected: 2009 | HbA1c 7-8: 25.3%  HbA1c > 8.0: 56.2% | Mean SBP (mmHg): 140.9  Mean DBP (mmHg): 84.5 | LDL-C ≥ 2.6(mmol/L): 86.6%  LDL-C < 2.6(mmol/L): 9.4% |
|  | (K. C. Tan et al., 2015) | Study design: Cross-Sectional  Sample size: 320  Study location: Melaka, Pulau Pinang  Year data collected: 2014 | HbA1c ≤6.5: 20.9%  HbA1c > 6.5: 79.1%  Mean HbA1c (%): 7.78 | n/a | n/a |
|  | (B. H. Chew et al., 2015b) | Study design: Cross-Sectional  Sample size: 700  Study location: Selangor  Year data collected: 2012 | Mean HbA1c (%): 8.5 | Mean SBP (mmHg): 136.9  Mean DBP (mmHg): 79.2 | Mean LDL-C (mmol/L): 3.0 |
|  | (Sazlina et al., 2010) | Study design: Cross-Sectional  Sample size: 396  Study location: Kuala Lumpur, Selangor  Year data collected: 2005 | HbA1c<6.5: 15.6%  HbA1c  ≥ 6.5: 84.4%  Mean HbA1c (%): 8.3 | BP ≤ 130/80mmHg: 50.6%  BP >130/80mmHg: 49.4%  Mean SBP (mmHg): 130  Mean DBP (mmHg): 80 | LDL-C ≤2.6(mmol/L): 13.0%  LDL-C > 2.6(mmol/L): 87.0%  Median LDL-C (mmol/L): 3.8 |
|  | (Chan et al., 2005) | Study design: Cross-Sectional  Sample size: 517  Study location: Melaka  Year data collected: 2003 | HbA1c 7-8: 29.2%  HbA1c > 8.0: 53.6%  Mean HbA1c (%): 8.5 | BP < 130/80mmHg: 3.1%  BP ≥ 140/90mmHg: 67.7% | LDL-C ≤2.6(mmol/L): 37.1%  LDL-C > 2.6(mmol/L): 62.9%  Mean LDL-C (mmol/L): 3.4 |
|  | (Mallika et al., 2011) | Study design: Cross-Sectional  Sample size: 738  Study location: Sarawak  Year data collected: 2004 | HbA1c<6.5: 10.9%  HbA1c 6.5- 7.5: 13.96%  HbA1c > 7.5: 75.07% | n/a | LDL-C ≤2.6(mmol/L): 19.6%  LDL-C > 2.6(mmol/L): 80.4% |
|  | (Cheong et al., 2012) | Study design: Cross-Sectional  Sample size: 200  Study location: Selangor  Year data collected: 2009 | HbA1c<6.5: 10.2%  Median HbA1c (%): 7.7 | BP ≤ 130/80mmHg: 15.5  Mean SBP (mmHg): 142.0  Mean DBP (mmHg): 84.0 | LDL-C ≤2.6(mmol/L): 14.8%  Mean LDL-C (mmol/L): 3.9 |
|  | (Wong, 2005) | Study design: Cross-Sectional  Sample size: 1337  Study location: Sarawak  Year data collected: 2003 | n/a | BP > 140/90mmHg: 34.8%  Mean SBP (mmHg): 137.0  Mean DBP (mmHg): 85.0 | n/a |
|  | (How et al., 2011) | Study design: Cross-Sectional  Sample size: 212  Study location: Kuala Lumpur  Year data collected: 2006 | HbA1c <7: 23.6%  Mean HbA1c (%): 8.1 | BP < 130/80: 24.5%  Mean SBP (mmHg): 134.5  Mean DBP (mmHg): 78.6 | LDL-C ≤2.6(mmol/L): 26.2%  Mean LDL-C (mmol/L): 3.1 |
|  | (Mafauzy, 2005) | Study design: Cross-Sectional  Sample size: 438  Study location: Kedah, Kelantan, Kuala Lumpur, Melaka, Negeri Sembilan, Pahang, Perlis, Pulau Pinang, Selangor, Terengganu.  Year data collected: 2001 | HbA1c<6.5: 11%  HbA1c ≤6.5: 13%  HbA1c 6.6- 7.5: 21%  HbA1c 6.5- 7.5: 23%  HbA1c <7: 20%  HbA1c > 7.5: 67%  HbA1c 7-8: 19%  HbA1c > 8.0: 61%  Mean HbA1c (%): 8.8 | DBP ≥ 90mmHg: 40.9%  SBP ≥ 140mmHg: 55.9%  BP < 130/80mmHg: 17.5% | n/a |
|  | (Mastura et al., 2007) | Study design: Cross-Sectional  Sample size: 556  Study location: Negeri Sembilan, Selangor.  Year data collected: 2006 | Mean HbA1c (%): 8.4 | Mean SBP (mmHg): 134.9  Mean DBP (mmHg): 81.5 | n/a |
|  | (Wong et al., 2007) | Study design: Cross-Sectional  Sample size: 1031  Study location: Sarawak  Year data collected: 2002 | HbA1c ≤6.5: 28%  HbA1c > 6.5: 72% | SBP ≤ 130mmHg: 27.0%  SBP > 130mmHg: 77.0%  BP ≤ 130/80mmHg: 6%  BP >130/80mmHg: 94%  DBP ≤ 80mmHg: 12%  DBP > 80mmHg: 88% | LDL-C ≤2.6(mmol/L): 22.0%  LDL-C > 2.6(mmol/L): 78.0% |
|  | (Rabia & Khoo, 2007) | Study design: Cross-Sectional  Sample size: 200  Study location: Kuala Lumpur  Year data collected: 2004 | Mean HbA1c (%): 8.38 | n/a | LDL-C ≤2.6(mmol/L): 12.0%  LDL-C ≥2.6(mmol/L): 87.5%  Mean LDL-C (mmol/L): 3.227 |
|  | (S. F. Tan et al., 2015) | Study design: Cohort  Sample size: 504  Study location: Kuala Lumpur  Year data collected: 2005 | HbA1c<6.5: 14.6%  Mean HbA1c (%): 8.5 | BP < 130/80mmHg: 6.5%  Mean SBP (mmHg): 139.4  Mean DBP (mmHg): 83.6 | Mean LDL-C (mmol/L): 3.7 |
|  | (Azmawati & Siti Norbayah, 2014) | Study design: Cross-Sectional  Sample size: 121  Study location: Kuala Lumpur  Year data collected: 2013 | HbA1c ≤6.5: 24%  HbA1c 6.6- 8.0: 41.3%  HbA1c 6.6- 8.0:34.7%  Mean HbA1c (%): 8.06 | n/a | n/a |
|  | (Azlina Wati et al., 2016) | Study design: Cross-Sectional  Sample size: 464  Study location: Not specified  Year data collected: 2015 | HbA1c  ≥ 6.5: 81.1%  HbA1c<6.5: 18.9% | n/a | n/a |
|  | (Nurjasmine Aida et al., 2018) | Study Design: Cross-Sectional  Sample size: 166  Study Location: Kuala Lumpur  Year data collected: 2012 | Mean HbA1c (%): 7.7 | n/a | n/a |
|  | (Shibraumalisi et al., 2020) | Study Design: Cross-Sectional  Sample size: 447  Study Location: Selangor  Year data collected: 2018 | HbA1c<6.5: 24.6%  HbA1c > 6.5: 75.4%  Mean HbA1c (%): 8.12 | BP < 140/90mmHg: 57.72%  BP ≥ 140/90mmHg: 42.28% | n/a |
|  | (Swarna Nantha et al., 2017) | Study Design: Case control  Sample size: 404  Study Location: Negeri Sembilan  Year data collected: 2015 | Mean HbA1c (%): 7.7 | n/a | n/a |
|  | (Hassan et al., 2021) | Study Design: Cross-Sectional  Sample size: 25,062  Study Location: Kedah  Year data collected: 2008 | HbA1c ≤6.5: 27.3%  HbA1c > 6.5: 72.7% | n/a | Mean LDL-C (mmol/L): 3.1 |
|  | (Chew et al., 2012b) | Study Design: Cross-Sectional  Sample size: 56503  Study Location: Not specified  Year data collected: 2009 | HbA1c ≤6.5: 46.1%  HbA1c > 6.5: 53.9% | BP < 130/80mmHg: 23.5%  SBP < 130mmHg: 33.8%  DBP <80mmHg: 44.8% | LDL-C ≤2.6(mmol/L): 21.2%  LDL-C ≥2.6(mmol/L): 78.8% |
|  | (Chew et al., 2012a) | Study Design: Cross-Sectional  Sample size: 70,889  Study Location: Not specified  Year data collected: 2009 | HbA1c ≤6.5: 18.1%  HbA1c  ≥ 6.5: 81.9%  HbA1c <7: 30.9%  HbA1c ≥7: 69.1%  Mean HbA1c (%): 8.3 | BP < 130/80mmHg: 23.5%  BP ≥130/80mmHg: 76.5%  Mean SBP (mmHg): 136.7  Mean DBP (mmHg): 78.8 | LDL-C ≤2.6(mmol/L): 31.0%  Mean LDL-C (mmol/L): 3.19 |
|  | (Tan et al., 2008) | Study Design: Cross-Sectional  Sample size: 196  Study Location: Sarawak  Year data collected: 2005 | HbA1c ≤ 7.0: 26%  HbA1c 7.0-8.0: 22%  HbA1c 8.0-9.0: 12%  HbA1c > 9.0: 9%  Mean HbA1c (%): 7.4 | BP ≤ 130/80mmHg: 32%  BP 130/80 - 140/90mmHg: 23%  BP 140/90 - 160/100mmHg: 31%  BP > 160/100mmHg: 13%  Mean SBP (mmHg): 142  Mean DBP (mmHg): 83 | LDL-C ≤2.6(mmol/L): 56%  LDL-C ≥2.6(mmol/L): 36%  Mean LDL-C (mmol/L): 2.4 |
|  | (Alias et al., 2023) | Study Design: Cross-Sectional  Sample size: 506  Study Location: Not specified  Year data collected: 2019 | HbA1c ≤ 7: 15.2%  Mean HbA1c (%): 9.29 | n/a | n/a |
|  | (Chew et al., 2013a) | Study Design: Cross-Sectional  Sample size: 57780  Study Location: Not specified  Year data collected: 2009 | HbA1c ≤6.5: 47.4%  HbA1c ≤ 7: 33.4%  Mean HbA1c (%): 8.37 | n/a | LDL-C ≤2.6(mmol/L): 20.8%  Mean LDL-C (mmol/L): 3.22 |
|  | (Chew et al., 2014) | Study Design: Cross-Sectional  Sample size: 20,481  Study Location: Negeri Sembilan, Perak, Selangor.  Year data collected: 2008 | HbA1c<6.5: 17.9%  HbA1c <7: 30.1%  Mean HbA1c (%): 8.0 | n/a | n/a |
|  | (Chew et al., 2010) | Study Design: Cross-Sectional  Sample size: 20,646  Study Location: Negeri Sembilan, Selangor  Year data collected: 2008 | n/a | BP ≤ 130/80mmHg: 41.4  BP >130/80mmHg: 58.6%  Mean SBP (mmHg): 137.7  Mean DBP (mmHg): 80.1 | n/a |
|  | (Azimah et al., 2010) | Study Design: Cross-Sectional  Sample size: 110  Study Location: Kuala Lumpur  Year data collected: 2008 | HbA1c<6.5: 45.5%  HbA1c  ≥ 6.5: 54.5% | n/a | n/a |
|  | (Rohana et al., 2007) | Study Design: Cross-Sectional  Sample size: 300  Study Location: Kelantan  Year data collected: 2005 | HbA1c <7: 13.7%  HbA1c ≥7: 86.3%  Mean HbA1c (%): 9.01 | Mean SBP (mmHg): 131.75  Mean DBP (mmHg): 82.11 | n/a |
|  | (Yudin et al., 2017) | Study Design: Cross-Sectional  Sample size: 234  Study Location: Kelantan  Year data collected: 2013 | Mean HbA1c (%): 7.92 | n/a | LDL-C <2.6(mmol/L): 37.6%  LDL-C ≥ 2.6(mmol/L): 62.4%  Mean LDL-C (mmol/L): 2.9 |
|  | (Tajudin et al., 2020) | Study Design: Cross-Sectional  Sample size: 22  Study Location: Johor  Year data collected: 2018 | Mean HbA1c (%): 8.92 | n/a | Mean LDL-C (mmol/L): 2.54 |
|  | (Hui Ng et al., 2012) | Study Design: Cross-Sectional  Sample size: 75  Study Location: Not specified  Year data collected: 2011 | HbA1c ≤6.5: 14.3%  HbA1c > 6.5: 85.7%  Mean HbA1c (%): 7.9 | n/a | n/a |
|  | (Singh et al., 2018) | Study Design: Cross-Sectional  Sample size: 334  Study Location: Negeri Sembilan  Year data collected: 2015 | Mean HbA1c (%): 7.8 | n/a | n/a |
|  | (Norma et al., 2010) | Study Design: Cohort  Sample size: 307  Study Location: Negeri Sembilan  Year data collected: 2007 | HbA1c ≤6.5: 22.2%  Mean HbA1c (%): 8.4 | BP < 130/80mmHg: 42.3%  Mean SBP (mmHg): 135.3  Mean DBP (mmHg): 82.7 | n/a |
|  | (Wong et al., 2020) | Study Design: Cross-Sectional  Sample size: 162  Study Location: Selangor  Year data collected: 2017 | HbA1c ≤6.5: 19.5%  HbA1c > 6.5: 80.5% | BP > 135/75mmHg: 82.1%  BP ≤ 135/75mmHg: 17.9% | LDL-C ≤2.6(mmol/L): 19.4%  LDL-C ≥2.6(mmol/L): 80.6% |
|  | (Ali et al., 2024) | Study design: Cross-Sectional  Sample size: 493  Study Location: Melaka  Year data collected: 2021 | Median HbA1c (%): 7.4 | BP < 140/80mmHg: 19.1% | n/a |
|  | (Ang et al., 2024) | Study design: Cross-Sectional  Sample size: 221,769  Study location:  Johor, Kedah, Kelantan, Melaka, Negeri Sembilan, Pahang, Perak, Perlis, Pulau Pinang,  Sabah, Sarawak, Selangor, Terengganu, Kuala Lumpur, Labuan, Putrajaya.  Year data collected: 2012, 2016, 2019 | 2012 HbA1c <7: 40.4%  2016 HbA1c <7: 42.1%  2019 HbA1c <7: 45.3% | n/a | n/a |
|  | (Mastura et al., 2011) | Study Design: Cross-Sectional  Sample size: 70,889  Study Location:  Kedah, Kelantan, Kuala Lumpur, Melaka, Negeri Sembilan, Pahang, Perak, Putrajaya, Selangor, Terengganu  Year data collected: 2009 | HbA1c<6.5: 18.1%  HbA1c <7: 30.9%  Mean HbA1c (%): 8.34 | SBP ≤ 130mmHg: 45%  DBP ≤ 80mmHg: 63.9%  BP ≤ 130/80mmHg: 38.2%  Mean SBP (mmHg): 136.72  Mean DBP (mmHg): 78.76 | LDL-C ≤2.6(mmol/L): 31.0%  Mean LDL-C (mmol/L): 3.19 |
|  | (Lee et al., 2013) | Study Design: Cross-Sectional  Sample size: 70,092  Study Location: Not specified  Year data collected: 2009 | HbA1c<6.5: 18.1% | BP ≤ 130/80mmHg: 23.5% | LDL-C ≤2.6(mmol/L): 31.0% |
|  | (Wan et al., 2021b) | Study Design: Cohort  Sample size: 17,592  Study Location: Negeri Sembilan  Year data collected: 2013 | 2013 Mean HbA1c <7: 38.6%  2014 Mean HbA1c <7: 40.4%  2015 Mean HbA1c <7: 40.7%  2016 Mean HbA1c <7: 42.6%  2017 Mean HbA1c <7: 39.4%  2013 Mean HbA1c (%): 8.03  2014 Mean HbA1c (%): 7.94  2015 Mean HbA1c (%): 7.95  2016 Mean HbA1c (%): 7.90  2017 Mean HbA1c (%): 8.04 | n/a | n/a |
|  | (Wan et al., 2021c) | Study Design: Cohort  Sample size: 18,341  Study Location: Negeri Sembilan  Year data collected: 2015 | Individualized Target HbA1c:  HbA1c ≤ 6.5: 29.6%  HbA1c < 7.0: 43.1%  HbA1c ≤ 8.0: 63.5%    Mean HbA1c (%): 7.88 | Individualized BP target:  BP < 130/80mmHg: 19.7%  BP ≥ 130/80mmHg: 80.3%  BP < 140/80mmHg: 36.1%  BP ≥ 140/80mmHg: 63.9mmHg  Mean SBP (mmHg): 134.85  Mean DBP (mmHg): 78.26 | Individualized LDL-C target:  LDL-C <2.6(mmol/L): 33.9%  LDL-C ≥ 2.6(mmol/L): 66.1%  LDL-C <1.8(mmol/L): 8.4%  LDL-C ≥ 1.8(mmol/L): 91.6%  LDL-C <1.4(mmol/L): 2.7%  LDL-C ≥ 1.4(mmol/L): 97.3%  Mean LDL-C (mmol/L): 2.91 |
|  | (Nor Shazwani et al., 2010) | Study Design: Cross-Sectional  Sample size: 132  Study Location: Kuala Lumpur  Year data collected: 2009 | HbA1c > 6.5: 72.2%  HbA1c<6.5: 27.8% | n/a | n/a |
|  | (Ahmad et al., 2014) | Study Design: Cross-Sectional  Sample size: 557  Study Location: Selangor  Year data collected: 2008 | HbA1c<6.5: 23%  Mean HbA1c (%): 8.04 | n/a | n/a |
|  | (Chew et al., 2011) | Study Design: Cross-Sectional  Sample size: 212  Study Location: Kuala Lumpur  Year data collected: 2009 | HbA1c ≤ 7: 30.3  Mean HbA1c (%): 8.10 | n/a | n/a |
|  | (Chew et al., 2018) | Study Design: Cross-Sectional  Sample size: 338  Study Location: Selangor  Year data collected: 2016 | HbA1c <7: 30.3%  HbA1c ≥7: 69.7%  Mean HbA1c (%): 8.30 | BP ≥ 140/80mmHg: 43%  BP > 140/90mmHg: 57%  Mean SBP (mmHg): 136.7  Mean DBP (mmHg): 76.4 | LDL-C <2.6(mmol/L): 44.2%  LDL-C ≥ 2.6(mmol/L): 55.8%  Mean LDL-C (mmol/L): 2.8 |
|  | (Chew et al., 2013b) | Study Design: Cross-Sectional  Sample size: 70,889  Study Location: Not specified  Year data collected: 2009 | HbA1c ≤6.5: 18.13%  HbA1c > 6.5: 81.87%  Mean HbA1c (%): 8.35 | BP < 130/80mmHg: 23.5%  BP ≥130/80mmHg: 76.5%  Mean SBP (mmHg): 136.74  Mean DBP (mmHg): 78.76 | LDL-C ≤2.6(mmol/L): 31.02%  LDL-C > 2.6(mmol/L): 68.98%  Mean LDL-C (mmol/L): 3.187 |
|  | (Lim et al., 2010) | Study Design: Cross-Sectional  Sample size: 132  Study Location: Kuala Lumpur  Year data collected: 2009 | HbA1c 3.5 - 6.5: 29.3%  Mean HbA1c (%): 8.25 | n/a | n/a |
|  | (Ministry of Health Malaysia, 2013) | Study Design: Report  Sample size: 653,326  Study Location: Johor, Kedah, Kelantan, Kuala Lumpur, Labuan, Melaka, Negeri Sembilan, Pahang, Perak, Perlis, Pulau Pinang, Putrajaya, Sabah, Sarawak, Selangor, Terengganu.  Year data collected: 2009 | 2009 HbA1c<6.5: 19.4%  2010 HbA1c<6.5: 24.8%  2011 HbA1c<6.5: 22.6%  2012 HbA1c<6.5: 23.8%  2009 HbA1c<7.0: 31.3%  2010 HbA1c<7.0: 38.9%  2011 HbA1c<7.0: 35.3%  2012 HbA1c<7.0: 37.9%  2009 HbA1c<8.0: 52.9%  2010 HbA1c<8.0: 58.6%  2011 HbA1c<8.0: 55.2%  2012 HbA1c<8.0: 57.5%  2009 HbA1c<10.0: 21.1%  2010 HbA1c<10.0: 18.1%  2011 HbA1c<10.0: 20.2%  2012 HbA1c<10.0: 19.4%  2009 Mean HbA1c (%): 8.30  2010 Mean HbA1c (%): 8.00  2011 Mean HbA1c (%): 8.20  2012 Mean HbA1c (%): 8.10 | 2009 SBP ≤ 130mmHg: 48.7%  2010 SBP ≤ 130mmHg: 52.6%  2011 SBP ≤ 130mmHg: 49.0%  2012 SBP ≤ 130mmHg: 47.6%  2009 BP ≤ 130/80mmHg: 41.2%  2010 BP ≤ 130/80mmHg: 45.0%  2011 BP ≤ 130/80mmHg: 42.0%  2012 BP ≤ 130/80mmHg: 40.9%  2009 DBP ≤ 80: 64.5%  2010 DBP ≤ 80: 67.1%  2011 DBP ≤ 80: 66.2%  2012 DBP ≤ 80: 67.1%  2009 Mean SBP (mmHg): 136.0  2010 Mean SBP (mmHg): 134.4  2011 Mean SBP (mmHg): 135.4  2012 Mean SBP (mmHg): 135.5  2009 Mean DBP (mmHg): 79.5  2010 Mean DBP (mmHg): 79.5  2011 Mean DBP (mmHg): 79.1  2012 Mean DBP (mmHg): 78.4 | 2009 LDL-C ≤2.6(mmol/L): 30.6%  2010 LDL-C ≤2.6(mmol/L): 33.6%  2011 LDL-C ≤2.6(mmol/L): 34.5%  2012 LDL-C ≤2.6(mmol/L): 37.8%  2009 Mean LDL-C (mmol/L): 3.2  2010 Mean LDL-C (mmol/L): 3.2  2011 Mean LDL-C (mmol/L): 3.2  2012 Mean LDL-C (mmol/L): 3.1 |
|  | (Abdullah et al., 2019) | Study Design: Cross-Sectional  Sample size: 200  Study Location: Selangor  Year data collected: 2017 | Mean HbA1c (%): 7.6 | n/a | n/a |
|  | (Ministry of Health Malaysia, 2009) | Study Design: Report  Sample size: 20,481  Study Location: Kedah, Kelantan, Kuala Lumpur, Negeri Sembilan, Pahang, Perak, Selangor, Terengganu  Year data collected: 2008 | Mean HbA1c (%): 8.39  Median HbA1c (%): 7.9 | SBP ≤ 130mmHg: 51.3%  SBP > 130mmHg: 48.7%  BP >130/80mmHg: 57.2%  BP ≤ 130/80mmHg: 42.8%  DBP ≤ 80mmHg: 64.5%  DBP > 80mmHg: 35.5%  Mean SBP (mmHg): 136.17  Median SBP (mmHg): 130.0  Mean DBP (mmHg): 81.04  Median DBP (mmHg): 80 | LDL-C ≤2.6(mmol/L): 30.7%  LDL-C > 2.6(mmol/L): 69.3%  Mean LDL-C (mmol/L): 3.21  Median LDL-C (mmol/L): 3.20 |
|  | (Ministry of Health Malaysia, 2010) | Study Design: Report  Sample size: 70,889  Study Location: Kedah, Kelantan, Kuala Lumpur, Melaka, Negeri Sembilan, Pahang, Perak, Putrajaya, Selangor, Terengganu  Year data collected: 2009 | HbA1c <6.5: 18.1%  HbA1c <7.0: 30.9%  Mean HbA1c (%): 8.34  Median HbA1c (%): 8.0 | BP >130/80mmHg: 61.8%  BP ≤ 130/80mmHg: 38.3%  SBP > 130mmHg: 55%  SBP ≤ 130mmHg: 45%  DBP ≤ 80mmHg: 63.9%  DBP > 80mmHg: 36.1%  Mean SBP (mmHg): 136.72  Median SBP (mmHg): 134  Mean DBP (mmHg): 78.76  Median DBP (mmHg): 80 | LDL-C ≤2.6(mmol/L): 31.0%  LDL-C > 2.6(mmol/L): 69.0%  Mean LDL-C (mmol/L): 3.19  Median LDL-C (mmol/L): 3.00 |
|  | (How et al., 2012) | Study Design: Cross-Sectional  Sample size: 20,646  Study Location: Negeri Sembilan, Perak, Selangor  Year data collected: 2008 | HbA1c <6.5: 18.0%  HbA1c <7.0: 30.3%  Mean HbA1c (%): 8.36 | SBP < 130mmHg: 31.04%  DBP <80mmHg: 31.20%  BP < 130/80mmHg: 16.32% | LDL-C <2.6(mmol/L): 30.1% |
|  | (Ministry of Health Malaysia, 2021) | Study Design: Report  Sample size: 1,687,384  Study Location: Johor, Kedah, Kelantan, Kuala Lumpur, Labuan, Melaka, Negeri Sembilan, Pahang, Perak, Perlis, Pulau Pinang, Putrajaya, Sabah, Sarawak, Selangor, Terengganu.  Year data collected: 2020 | HbA1c ≤6.5: 34.35%  HbA1c ≤ 7.0: 43.74%  HbA1c ≤8.0: 63.04%  HbA1c  ≥ 10.0: 14.95%  Mean HbA1c (%): 7.8 | BP ≤ 135/75mmHg: 26.33%  BP >135/75mmHg: 73.67%  SBP ≤135mmHg: 48.14%  DBP ≤75mmHg: 41.65%  DBP ≥76mmHg: 58.35%  Mean SBP (mmHg): 136.9  Mean DBP (mmHg): 77.6 | LDL-C ≤2.6(mmol/L): 45.68%  LDL-C > 2.6(mmol/L): 54.30%  Mean LDL-C (mmol/L): 2.9 |
|  | (Mafauzy et al., 1999) | Study Design: Cross-Sectional  Sample size: 2508  Study Location: Kelantan  Year data collected: 1997 | n/a | Mean SBP (mmHg): 137.1  Mean DBP (mmHg): 81.2 | Mean LDL-C (mmol/L): 4.3 |
|  | (B. H. Chew et al., 2015a) | Study Design: Cross-Sectional  Sample size: 175  Study Location: Kuala Lumpur  Year data collected: 2006 | HbA1c ≤ 7:23.6%  Mean HbA1c (%): 8.15 | n/a | n/a |
|  | (Ministry of Health Malaysia, 2020) | Study Design: Cross-Sectional  Sample size: 1602882  Study Location: Johor, Kedah, Kelantan, Melaka, Negeri Sembilan, Pahang, Perak, Perlis, Pulau Pinang,  Sabah, Sarawak, Selangor, Terengganu, Kuala Lumpur, Labuan, Putrajaya.  Year data collected: 2013, 2014, 2015, 2016, 2017, 2018, 2019. | 2013 HbA1c ≤6.5: 30.35%  2014 HbA1c ≤6.5: 33.09%  2015 HbA1c ≤6.5: 29.62%  2016 HbA1c ≤6.5: 30.60%  2017 HbA1c ≤6.5: 30.56%  2018 HbA1c ≤6.5: 31.42%  2019 HbA1c ≤6.5: 32.41%  2013 HbA1c ≤7.0: 38.87%  2014 HbA1c ≤7.0: 41.43%  2015 HbA1c ≤7.0: 38.07%  2016 HbA1c ≤7.0: 38.96%  2017 HbA1c ≤7.0: 39.36%  2018 HbA1c ≤7.0: 40.02%  2019 HbA1c ≤7.0: 41.26%  2013 HbA1c ≤8.0: 19.35%  2014 HbA1c ≤8.0: 18.14%  2015 HbA1c ≤8.0: 19.69%  2016 HbA1c ≤8.0: 19.38%  2017 HbA1c ≤8.0: 18.63%  2018 HbA1c ≤8.0: 18.13%  2019 HbA1c ≤8.0: 17.05%  2013 Mean HbA1c (%): 8.1  2014 Mean HbA1c (%): 8.0  2015 Mean HbA1c (%): 8.1  2016 Mean HbA1c (%): 8.1  2017 Mean HbA1c (%): 8.0  2018 Mean HbA1c (%): 8.0  2019 Mean HbA1c (%): 7.9 | 2013 SBP ≤135mmHg: 55.3%  2014 SBP ≤135mmHg: 55.4%  2015 SBP ≤135mmHg: 54.0%  2016 SBP ≤135mmHg: 53.3%  2017 SBP ≤135mmHg: 51.9%  2018 SBP ≤135mmHg: 52.6%  2019 SBP ≤135mmHg: 52.2%  2013 DBP ≤75mmHg: 39.0%  2014 DBP ≤75mmHg: 40.6%  2015 DBP ≤75mmHg: 42.2%  2016 DBP ≤75mmHg: 42.8%  2017 DBP ≤75mmHg: 42.2%  2018 DBP ≤75mmHg: 43.0%  2019 DBP ≤75mmHg: 44.5%  2013 BP ≤ 135/75mmHg: 27.4%  2014 BP ≤ 135/75mmHg: 28.3%  2015 BP ≤ 135/75mmHg: 28.9%  2016 BP ≤ 135/75mmHg: 29.2%  2017 BP ≤ 135/75mmHg: 28.3%  2018 BP ≤ 135/75mmHg: 28.9%  2019 BP ≤ 135/75mmHg: 29.3%  2013 Mean SBP (mmHg): 135.1  2014 Mean SBP (mmHg): 134.8  2015 Mean SBP (mmHg): 135.4  2016 Mean SBP (mmHg): 135.4  2017 Mean SBP (mmHg): 135.8  2018 Mean SBP (mmHg): 135.4  2019 Mean SBP (mmHg): 135.4  2013 Mean DBP (mmHg): 77.7  2014 Mean DBP (mmHg): 77.4  2015 Mean DBP (mmHg): 77.1  2016 Mean DBP (mmHg): 77.1  2017 Mean DBP (mmHg): 77.3  2018 Mean DBP (mmHg): 77.1  2019 Mean DBP (mmHg): 76.9 | 2013 LDL-C ≤2.6(mmol/L): 37.3%  2014 LDL-C ≤2.6(mmol/L): 39.2%  2015 LDL-C ≤2.6(mmol/L): 40.2%  2016 LDL-C ≤2.6(mmol/L): 42.7%  2017 LDL-C ≤2.6(mmol/L): 43.2%  2018 LDL-C ≤2.6(mmol/L): 44.9%  2019 LDL-C ≤2.6(mmol/L): 45.1%  2013 Mean LDL-C (mmol/L): 3.1  2014 Mean LDL-C (mmol/L): 3.0  2015 Mean LDL-C (mmol/L): 3.0  2016 Mean LDL-C (mmol/L): 3.0  2017 Mean LDL-C (mmol/L): 3.0  2018 Mean LDL-C (mmol/L): 2.9  2019 Mean LDL-C (mmol/L): 2.9 |
|  | (Ministry of Health Malaysia, 2024) | Study design: Report  Sample size: 220,645  Study location: Johor, Kedah, Kelantan, Melaka, Negeri Sembilan, Pahang, Perak, Perlis, Pulau Pinang,  Sabah, Sarawak, Selangor, Terengganu, Kuala Lumpur, Labuan, Putrajaya.  Year data collected: 2022, 2023 | 2022 HbA1c ≤6.5: 34.9%  2023 HbA1c ≤6.5: 34.4%  2022 Mean HbA1c (%): 7.8  2023 Mean HbA1c (%): 7.7 | 2022 SBP ≤135mmHg: 49%  2023 SBP ≤135mmHg: 50.9%  2022 DBP ≤75mmHg: 43%  2023 DBP ≤75mmHg: 43.9%  2022 ≤135/75mmHg: 27.1%  2023 ≤135/75mmHg: 28.2%  2022 Mean SBP (mmHg): 136.2  2023 Mean SBP (mmHg): 135  2022 Mean DBP (mmHg): 77.2  2023 Mean DBP (mmHg): 77 | 2022 LDL-C ≤2.6(mmol/L): 50.3%  2023 LDL-C ≤2.6(mmol/L): 52.7%  2022 Mean LDL (mmol/L): 2.8  2023 Mean LDL (mmol/L): 2.6 |

n/a: not available.

**List of publications included in the scoping review**

1. Ab Rahman, N., Lim, M. T., Thevendran, S., Ahmad Hamdi, N., & Sivasampu, S. (2022). Medication Regimen Complexity and Medication Burden Among Patients With Type 2 Diabetes Mellitus: A Retrospective Analysis. *Frontiers in Pharmacology*.
2. Abdullah, A., Ng, C. J., Liew, S. M., Ambigapathy, S., V, P., & Chinna, K. (2020). Prevalence of limited health literacy and its associated factors in patients with type 2 diabetes mellitus in Perak, Malaysia: a cross-sectional study. *BMJ open*.
3. Abdullah, F. i., Han, T. M., Mat Nor, M. B., Mohd Aznan, M. A., & Ismail, I. Z. (2017). Prevalence of hypertension and glycaemic control in adult type-2 diabetes patients: A preliminary retrospective cohort study in Kuantan, Pahang, Malaysia. *International Medical Journal Malaysia*.
4. Abdullah, N. A., Ismail, S., Ghazali, S. S., Juni, M. H., Kadir, H., & Aziz, N. R. A. (2019). Predictors of Good Glycemic Controls Among Type 2 Diabetes Mellitus Patients in Two Primary Health Clinics, Kuala Selangor. *Malaysian Journal of Medicine and Health Sciences*.
5. Ahmad, N. S., Islahudin, F., & Paraidathathu, T. (2014). Factors associated with good glycemic control among patients with type 2 diabetes mellitus. *Journal of Diabetes Investigation*.
6. Ali, H., May, J. M., & Rashid, A. A. (2024). Prevalence of Diabetic Peripheral Neuropathy Among Type 2 Diabetes mellitus and Its Associated Factors in a Primary Care Clinic in Malacca: A Cross-sectional Study. *Malaysian Journal of Medicine & Health Sciences*, *20*(5).
7. Alias, S. H., Teng, C. L., Devaraj, N. K., Amirrudin, S., Latib, N. R. A., Chong, F. Y., Yussof, S., Din, M. D. M., & Lim, P. W. (2023). Prevalence and associated factors of lipohypertrophy in insulin-injected patients with diabetes in selected primary care clinics in Peninsular Malaysian: A cross-sectional study. *Malaysian Family Physician*.
8. Ang, S. H., Lim, L.-L., Mustapha, F. I., Ahmad, E., & Rampal, S. (2024). Association between sex, age, temporal trends, and glycemic control of 221,769 adults with type 2 diabetes in a multi-ethnic middle-income Asian country. *Diabetes research and clinical practice*, *220*, 111976.
9. Azimah, M., Radzniwan, R., Zuhra, H., & Khairani, O. (2010). Have we done enough with diabetic education? A pilot study. *Malaysian Family Physician*.
10. Azlina Wati, N., Mohd Ariff, F., & Sakinah, I. (2016). Self-perceived anxiety symptoms and its associated factors among type 2 diabetic patients in rural communities of Malaysia. *Journal of Clinical and Health Sciences*.
11. Azmawati, M. N., & Siti Norbayah, Y. (2014). Assessment of physical inactivity and its associated factors among type 2 diabetes mellitus patients in a university primary clinic in Kuala Lumpur. *Malaysian journal of nutrition*.
12. Azura, M. S., Adibah, H. I., & Juwita, S. (2012). Risk factor of peripheral neuropathy among newly diagnosed type 2 diabetic patients in primary care clinic. *International Journal of Collaborative Research on Internal Medicine and Public Health*.
13. Bujang, M. A., Mohan, K., Mohd Hatta, N. K. B., Baharum, N., & Ismail, M. (2021). Quality of life and its associated factors among type 2 diabetes patients in malaysian primary health care. *International Medical Journal*.
14. Chamhuri, N. H., Mohd Tohit, N., Azzeri, A., Chamhuri, N., & M Alias, S. R. (2022). Age and fasting blood sugar levels are associated factors for mindful eating among Type 2 diabetes mellitus patients during COVID-19 pandemic confinement. *PloS one*.
15. Chan, G. C. (2005). Type 2 diabetes mellitus with hypertension at primary healthcare level in Malaysia: are they managed according to guidelines? *Singapore medical journal*.
16. Chan, G. C., Ghazali, O., & Khoo, E. M. (2005). Management of type 2 diabetes mellitus: Is it in accordance with the guidelines? *Medical Journal of Malaysia*.
17. Chen, L. L., Yaacob, S., & Ming, T. K. (2022). Insulin Therapy Refusal among Insulin-naive Type II Diabetes Mellitus Patients in Segamat. *Malaysian Journal of Medicine and Health Sciences*.
18. Cheong, A. T., Ahmad, Z., & Chew, B. H. (2012). Metabolic control and cardiovascular risk factors among type 2 diabetes in a primary care clinic. *Malaysian Journal of Medicine and Health Sciences*.
19. Chew, B.-H., Hussain, H., & Supian, Z. A. (2021). Is therapeutic inertia present in hyperglycaemia, hypertension and hypercholesterolaemia management among adults with type 2 diabetes in three health clinics in Malaysia? a retrospective cohort study. *BMC family practice*.
20. Chew, B.-H., Mohd-Sidik, S., & Shariff-Ghazali, S. (2015). Negative effects of diabetes-related distress on health-related quality of life: An evaluation among the adult patients with type 2 diabetes mellitus in three primary healthcare clinics in Malaysia. *Health and quality of life outcomes*.
21. Chew, B. H., Ismail, M., Lee, P. Y., Taher, S. W., Haniff, J., Mustapha, F. I., & Bujang, M. A. (2012a). Determinants of uncontrolled dyslipidaemia among adult type 2 diabetes in Malaysia: The Malaysian Diabetes Registry 2009. *Diabetes research and clinical practice*.
22. Chew, B. H., Khoo, E. M., & Chia, Y. C. (2011). Does religious affiliation influence glycaemic control in primary care patients with type 2 diabetes mellitus? *Mental health in family medicine*.
23. Chew, B. H., Khoo, E. M., & Chia, Y. C. (2015a). Social support and glycemic control in adult patients with type 2 diabetes mellitus. *Asia-Pacific journal of public health*.
24. Chew, B. H., Lee, P. Y., Mastura, I., Cheong, A. T., Sri Wahyu, T., & Zaiton, A. (2014). Glycaemic control and treatment profile amongst 20646 adult type 2 diabetes mellitus: A descriptive report. *Pertanika Journal of Science & Technology*.
25. Chew, B. H., Mastura, I., Cheong, A. T., & Syed Alwi, S. A. R. (2010). Diabetic hypertensive control and treatment: A descriptive report from the audit diabetes control and management (ADCM) registry. *Malaysian Family Physician*.
26. Chew, B. H., Mastura, I., Shariff-Ghazali, S., Lee, P. Y., Cheong, A. T., Ahmad, Z., Taher, S. W., Haniff, J., Mustapha, F. I., & Bujang, M. A. (2012b). Determinants of uncontrolled hypertension in adult type 2 diabetes mellitus: an analysis of the Malaysian diabetes registry 2009. *Cardiovascular diabetology*.
27. Chew, B. H., Shariff-Ghazali, S., Lee, P. Y., Cheong, A. T., Mastura, I., Haniff, J., Bujang, M. A., Taher, S. W., & Mustapha, F. I. (2013a). Type 2 Diabetes Mellitus Patient Profiles, Diseases Control and Complications at Four Public Health Facilities- A Cross-sectional Study based on the Adult Diabetes Control and Management (ADCM) Registry 2009. *Medical Journal of Malaysia*.
28. Chew, B. H., Shariff Ghazali, S., Ismail, M., Haniff, J., & Bujang, M. A. (2013b). Age ≥60 years was an independent risk factor for diabetes-related complications despite good control of cardiovascular risk factors in patients with type 2 diabetes mellitus. *Experimental Gerontology*.
29. Chew, B. H., Sherina, M. S., & Hassan, N. H. (2015b). Association of diabetes-related distress, depression, medication adherence, and health-related quality of life with glycated hemoglobin, blood pressure, and lipids in adult patients with type 2 diabetes: A cross-sectional study. *Therapeutics and clinical risk management*.
30. Chew, B. H., Vos, R. C., Pouwer, F., & Rutten, G. E. H. M. (2018). The associations between diabetes distress and self-efficacy, medication adherence, self-care activities and disease control depend on the way diabetes distress is measured: Comparing the DDS-17, DDS-2 and the PAID-5. *Diabetes research and clinical practice*.
31. Chew, B. H., vos, R. C., Stellato, R. K., & Rutten, G. E. H. M. (2017). Diabetes-related distress and depressive symptoms are not merely negative over a 3-year period in Malaysian adults with type 2 diabetes mellitus receiving regular primary diabetes care. *Frontiers in Psychology*.
32. Chin, S. S., Lau, S. W., Lim, P. L., Wong, C. M., & Ujang, N. (2023). Medication adherence, its associated factors and implication on glycaemic control in patients with type 2 diabetes mellitus: A cross-sectional study in a Malaysian primary care clinic. *Malaysian Family Physician*.
33. Chin, Y. W., Lai, P. S. M., & Chia, Y. C. (2017). The validity and reliability of the English version of the diabetes distress scale for type 2 diabetes patients in Malaysia. *BMC family practice*.
34. Ching, S. M., Zakaria, Z. A., Paimin, F., & Jalalian, M. (2013). Complementary alternative medicine use among patients with type 2 diabetes mellitus in the primary care setting: A cross-sectional study in Malaysia. *BMC complementary and alternative medicine*.
35. Dhillon, H., Nordin, R. B., & Ramadas, A. (2019). Quality of life and associated factors among primary care Asian patients with type 2 diabetes mellitus. *International journal of environmental research and public health*.
36. Goh, S. S. L., Lai, P. S. M., Liew, S.-M., Tan, K. M., Chung, W. W., & Chua, S. S. (2020). Development of a PATIENT-Medication Adherence Instrument (P-MAI) and a HEALTHCARE PROFESSIONAL-Medication Adherence Instrument (H-MAI) using the nominal group technique. *PloS one*.
37. Gunggu, A., Thon, C. C., & Whye Lian, C. (2016). Predictors of Diabetes Self-Management among Type 2 Diabetes Patients. *Journal of diabetes research*.
38. Hashim, N. A., Ariaratnam, S., Salleh, M. R., Said, M. A., & Sulaiman, A. H. (2016). Depression and associated factors in patients with type 2 diabetes mellitus. *East Asian Archives of Psychiatry*.
39. Hassan, M. R., Jamhari, M. N., Hayati, F., Ahmad, N., Zamzuri, M. A. I. A., Nawi, A. M., Sharif, K. Y., Sufri, M., Ahmad, S. B., Ismail, N., Rahim, S. S. S. A., & Jeffree, M. S. (2021). Determinants of glycaemic control among type 2 diabetes mellitus patients in Northern State of Kedah, Malaysia: a cross-sectional analysis of 5 years national diabetes registry 2014-2018. *Pan African Medical Journal*.
40. How, C. B., Ai-Theng, C., Ahmad, Z., & Ismail, M. (2012). Men suffer more complications from diabetes than women despite similar glycaemic control and a better cardiovascular risk profile: The adcm study 2008. *Journal of Men's Health*.
41. How, C. B., Ming, K. E., & Chin, C. Y. (2011). Quality of care for adult type 2 Diabetes Mellitus at a University primary care centre in Malaysia. *International Journal of Collaborative Research on Internal Medicine and Public Health*.
42. Hui Ng, S., Chan, K. H., Lian, Z. Y., Chuah, Y. H., Waseem, A. N., & Kadirvelu, A. (2012). Reality vs illusion: Knowledge, attitude and practice among diabetic patients. *International Journal of Collaborative Research on Internal Medicine and Public Health*.
43. Husain, N. F., Yusoff, H. M., Hassan, N. M., & Aziz, A. A. (2023). Prevalence of Latent Tuberculosis Infection and its Associated Factors Among Diabetic Patients Availing Primary Health Care in Terengganu State, Malaysia. *Oman medical journal*.
44. Jamaluddin, J., & Mohamed Kamel, M. A. (2024). Underprescription of Fibrate Among Patients With Diabetic Retinopathy in Perak, Malaysia. *Cureus*.
45. Jusoh, Z., Tohid, H., Omar, K., Muhammad, N. A., & Ahmad, S. (2018). Clinical and Sociodemographic Predictors of the Quality of Life among Patients with Type 2 Diabetes Mellitus on the East Coast of Peninsular Malaysia. *Malaysian Journal of Medical Sciences*.
46. Kaur, G., Tee, G. H., Ariaratnam, S., Krishnapillai, A. S., & China, K. (2013). Depression, anxiety and stress symptoms among diabetics in Malaysia: A cross sectional study in an urban primary care setting. *BMC family practice*.
47. Lai, P. S. M., Sellappans, R., & Chua, S. S. (2020). Reliability and Validity of the M-MALMAS Instrument to Assess Medication Adherence in Malay-Speaking Patients with Type 2 Diabetes. *Pharmaceutical medicine*.
48. Lee, C. L., Chee, W. S. S., Arasu, K., Kwa, S. K., & Mohd Ali, S. Z. (2019). Diabetes literacy and knowledge among patients with type 2 diabetes mellitus attending a primary care clinic in Seremban, Malaysia. *Malaysian journal of nutrition*.
49. Lee, P. Y., Cheong, A. T., Zaiton, A., Mastura, I., Chew, B.-H., Sazlina, S. G., Adam, B. M., Syed Alwi, S. A. R., Jamaiyah, H., & SriWahyu, T. (2013). Does Ethnicity Contribute to the Control of Cardiovascular Risk Factors Among Patients With Type 2 Diabetes? *Asia Pacific Journal of Public Health*.
50. Lee, P. Y., Salim, H. S., Cheng, Y. G., Zainuddin, Z., Singh, H., & Loh, K. W. (2022). The proportion of undiagnosed diabetic peripheral neuropathy and its associated factors among patients with T2DM attending urban health clinics in Selangor. *Malaysian Family Physician*.
51. Lee, Y. L., Lim, Y. M. F., Law, K. B., & Sivasampu, S. (2020). Intra-cluster correlation coefficients in primary care patients with type 2 diabetes and hypertension. *Trials*.
52. Leelavathi, M., Azimah, M. N., Kharuddin, N. F., & Tzar, M. N. (2013). Prevalence of toenail onychomycosis among diabetics at a primary care facility in Malaysia. *Southeast Asian Journal of Tropical Medicine and Public Health*.
53. Lim, C. J., Shahar, S., Yahya, H. M., Teh, S. C., Mohd Nor, N. S., Lim, H. C., Mohd Zaki, M. F., Sallehuddin, D., & Mukhsan, N. (2010). Tahap Pengetahuan Pemakanan dan Kesedaran Kesihatan di Kalangan Pesakit Diabetes Mellitus di Klinik Kesihatan, Cheras, Kuala Lumpur, Malaysia (Level of Nutritional Knowledge and Health Awareness Among Diabetes Mellitus Patients at Cheras Health Clinic, Kuala Lumpur, Malaysia). *Sains Malaysiana*.
54. Lim, M. T., Ab Rahman, N., Teh, X. R., Chan, C. L., Thevendran, S., Ahmad Hamdi, N., Lim, K. K., & Sivasampu, S. (2021). Optimal cut-off points for adherence measure among patients with type 2 diabetes in primary care clinics: a retrospective analysis. *Therapeutic advances in chronic disease*.
55. Lim, Y. M. F., Ang, S. H., Nasir, N. H., Ismail, F., Ismail, S. A., & Sivasampu, S. (2019). Clinic and patient variation in intermediate clinical outcomes for type 2 diabetes: a multilevel analysis. *BMC family practice*.
56. Mafauzy, M. (2005). Diabetes control and complications in private primary healthcare in Malaysia. *Medical Journal of Malaysia*.
57. Mafauzy, M., Mokhtar, N., Wan Mohamad, W. B., & Musalmah, M. (1999). Diabetes mellitus and associated cardiovascular risk factors in North-East Malaysia. *Asia-Pacific journal of public health*.
58. Mahmood, M. I., Daud, F., & Ismail, A. (2016). Glycaemic control and associated factors among patients with diabetes at public health clinics in Johor, Malaysia. *Public health*.
59. Mallika, P., Lee, P., Cheah, W., Wong, J., Syed Alwi, S., Nor Hayati, H., & Tan, A. (2011). Risk factors for diabetic retinopathy in diabetics screened using fundus photography at a primary health care setting in East Malaysia. *Malaysian Family Physician*.
60. Mastura, H. I., Mimi, O., Piterman, L., Teng, C. L., & Wijesinha, S. (2007). Self-monitoring of blood glucose among diabetes patients attending government health clinics. *Medical Journal of Malaysia*.
61. Mastura, I., Chew, B. H., Lee, P. Y., Cheong, A. T., Sazlina, S.-G., Jamaiyah, H., syed abdul rahman, s. a., Taher, S., & Zaiton, A. (2011). Control and Treatment Profiles of 70,889 Adult Type 2 Diabetes Mellitus Patients in Malaysia - A Cross Sectional Survey in 2009. *International Journal of Collaborative Research on Internal Medicine & Public Health*.
62. Ministry of Health Malaysia. (2009). *Diabetes Registry Malaysia: Preliminary Report of An Audit Of Diabetes Control and Management (July-December 2008)*.
63. Ministry of Health Malaysia. (2010). *Diabetes Registry Malaysia: Report of An Audit Of Diabetes Control and Management (January-December 2009)*.
64. Ministry of Health Malaysia. (2013). *National Diabetes Registry Report: Volume 1, 2009-2012*.
65. Ministry of Health Malaysia. (2020). *National Diabetes Registry Report 2013-2019*.
66. Ministry of Health Malaysia. (2021). *National Diabetes Registry Report 2020*.
67. Ministry of Health Malaysia. (2024). *National Diabetes Registry Report 2023*.
68. Mohd Aznan, M. A., Khairidzan, M. K., Razman, M. R., & Fa’iza, A. (2018). Prevalence of diabetic retinopathy and its associated factors among diabetic patients in primary care clinics, Kuantan, Pahang. *International Medical Journal Malaysia*.
69. Moy Foong, M., & Yew Sheng, Q. (2019). Predicting the risk of chronic kidney disease among type 2 diabetes mellitus patients in a primary care setting: an evaluation of the QKidney model. *Malaysian Journal of Medicine and Health Sciences*.
70. Naserrudin, N. A., Jeffree, M. S., Kaur, N., Syed Abdul Rahim, S. S., & Ibrahim, M. Y. (2022). Diabetic retinopathy among type 2 diabetes mellitus patients in Sabah primary health clinics - Addressing the underlying factors. *PloS one*.
71. Nasir, N. F. M., Draman, N., Zulkifli, M. M., Muhamad, R., & Draman, S. (2022). Sleep Quality among Patients with Type 2 Diabetes: A Cross-Sectional Study in the East Coast Region of Peninsular Malaysia. *International journal of environmental research and public health*.
72. Nor Shazwani, M. N., Suzana, S., Hanis Mastura, Y., Lim, C. J., Teh, S. C., Mohd Fauzee, M. Z., Lim, H. C., Dahlia, S., & Norliza, M. (2010). Assessment of Physical Activity Level among Individuals with Type 2 Diabetes Mellitus at Cheras Health Clinic, Kuala Lumpur. *Malaysian journal of nutrition*.
73. Nordin, N., Hairon, S. M., Yaacob, N. M., Hamid, A. A., & Hassan, N. (2020). Effects of FamilyDoctor Concept and Doctor-Patient Interaction Satisfaction on Glycaemic Control among Type 2 Diabetes Mellitus Patients in the Northeast Region of Peninsular Malaysia. *International journal of environmental research and public health*.
74. Nordin, N., Hairon, S. M., Yaacob, N. M., Hamid, A. A., Isa, S. A. M., & Hassan, N. (2021). Perceived quality of care among people with type 2 diabetes mellitus in the north east region of peninsular Malaysia. *BMC public health*.
75. Norma, S., Azmi, M. T., & Rotina, A. B. (2010). Factors that influence albuminuria among type 2 diabetes mellitus at primary healthcare clinics in Negeri Sembilan 2007. *Malaysian Journal of Community Health*.
76. Nurjasmine Aida, J., Noor Azimah, M., Aida, J., Saharuddin, A., & Noorlaili, T. (2018). Foot problem and Foot care practices among diabetic patients in a primary care clinic, Kuala Lumpur. *International Journal of Allied Health Sciences*.
77. Ong, J. J., Azmil, S. S., Kang, C. S., Lim, S. F., Ooi, G. C., Patel, A., & Mawardi, M. (2022). Foot care knowledge and self-care practices among diabetic patients in Penang: A primary care study. *Medical Journal of Malaysia*.
78. Papo, M., Tohid, H., Ahmad, S., Sumeh, A. S., Jamil, T. R., & Hamzah, Z. (2019). Self-care activities among patients with type 2 diabetes mellitus in Penampang, Sabah and its association with depression, anxiety and stress. *Malaysian Journal of Public Health Medicine*.
79. Rabia, K., & Khoo, E. M. (2007). Prevalence of peripheral arterial disease in patients with diabetes mellitus in a primary care setting. *Medical Journal of Malaysia*.
80. Rashid, A. A., Hamzah, Z., Eng, T. C., & Devaraj, N. K. (2020). Effects of social support and self-efficacy of glucose control among Malaysian type 2 diabetes mellitus patients in primary care. *Malaysian Journal of Medicine and Health Sciences*.
81. Rasid, S., Muthupalaniappen, L., & Jamil, A. (2020). Prevalence and factors associated with cutaneous manifestations of type 2 diabetes mellitus. *Clinical Diabetology*.
82. Rohana, D., Wan Norlida, W. l., Nor Azwany, Y., Mazlan, A., Zawiyah, D., Che Karrialudin, C. A., & Che Ghani, C. S. (2007). Economic evaluation of type 2 diabetes management at the Malaysian Ministry of Health primary care clinics, in Machang, Kelantan. *Malaysian Journal of Public Health Medicine*.
83. Samat, F., Samad, S., Rosnan, S. K., Kaur, J., Zulkefli, N. H., Rosli, N. A., Ismail, F. N., & Aziz, N. R. A. (2024). Usage of vildagliptin among patients with type 2 diabetes mellitus attending a public primary healthcare clinics in Kuala Selangor District, Selangor. *Medical Journal of Malaysia*.
84. Sazlina, S.-G., Lee, P. Y., Chan, Y. M., A Hamid, M. S., & Tan, N. C. (2020). The prevalence and factors associated with sarcopenia among community living elderly with type 2 diabetes mellitus in primary care clinics in Malaysia. *PloS one*.
85. Sazlina, S. G., Zailinawati, A. H., Zaiton, A., & Ong, I. (2010). A clinical audit on diabetes care in two urban public primary care clinics in Malaysia. *Malaysian Journal of Medicine and Health Sciences*.
86. Shibraumalisi, N. A., Mat Nasir, N., Md Yasin, M., & Isa, M. R. (2020). The association between health literacy and quality of life and its associated factors among adults with type 2 diabetes mellitus in public primary care clinic. *Journal of Clinical and Health Sciences*.
87. Singh, H. K. G., Mun, V. L. K., Barua, A., Ali, S. Z. M., & Swee, W. C. S. (2018). Application and validation of the weight efficacy lifestyle (WEL) questionnaire among type 2 diabetes mellitus patients in Malaysia. *Malaysian journal of nutrition*.
88. Swarna Nantha, Y., Puri, A., Mohamad Ali, S. Z., Suppiah, P., Che Ali, S. A., Ramasamy, B., & Ibrahim, I. M. (2017). Epidemiology of latent tuberculosis infection among patients with and without diabetes mellitus. *Family Practice*.
89. Syed Soffian, S. S., Ahmad, S. B., Chan, H.-K., Soelar, S. A., Abu Hassan, M. R., & Ismail, N. (2019). Management and glycemic control of patients with type 2 diabetes mellitus at primary care level in Kedah, Malaysia: A statewide evaluation. *PloS one*.
90. Tajudin, T. R., Harmal, N. S., Abdulshakur, Z., Yusof, A. H. K., Tay, C. L., Teoh, S. H., Rashid, A. A., Mohamad, F., Manap, A. H., & Devaraj, N. K. (2020). Establishment of a diabetic clinic and its effects on glycaemic control among diabetic patients in muar district, malaysia. *Sapporo Medical journal*.
91. Tan, F., Chan, G., Wong, J. S., & Rozario, F. (2008). Standard of care for type 2 diabetic patients in a public hospital general medical clinic: report of a self-audit. *The Medical journal of Malaysia*.
92. Tan, J. Y. H., & Ng, C. J. (2023). Prevalence and burden of diabetes mellitus-related symptoms in patients with type 2 diabetes mellitus: A cross-sectional study. *Malaysian Family Physician*.
93. Tan, K. C., Chan, G. C., Eric, H., Maria, A. I., Norliza, M. J., Oun, B. H., Sheerine, M. T., Wong, S. J., & Liew, S. M. (2015). Depression, anxiety and stress among patients with diabetes in primary care: A cross-sectional study. *Malaysian Family Physician*.
94. Tan, S. F., Chia, Y. C., & Chinna, K. (2015). Rate of decline of kidney function in patients with type 2 diabetes mellitus and the associated factors: a 10-year retrospective cohort study. *Asia-Pacific journal of public health*.
95. Tan, W. L., Asahar, S. F., & Harun, N. L. (2015). Insulin therapy refusal among type ii diabetes mellitus patients in Kubang Pasu district, Kedah, Malaysia. *Singapore medical journal*.
96. Tan, W. Y., & Ismail, M. (2020). Health literacy among adult type 2 diabetes mellitus (T2DM) patients in Klang Health District Malaysia. *Journal of Health and Translational Medicine*.
97. Tharek, Z., Ramli, A. S., Whitford, D. L., Ismail, Z., Mohd Zulkifli, M., Ahmad Sharoni, S. K., Shafie, A. A., & Jayaraman, T. (2018). Relationship between self-efficacy, self-care behaviour and glycaemic control among patients with type 2 diabetes mellitus in the Malaysian primary care setting. *BMC family practice*.
98. Wan, K. S., Hairi, N. N., Moy, F. M., Mustapha, F. I., Yusof, K. M., & Ali, Z. M. (2021a). Poorer Attainment of Hemoglobin A1C, Blood Pressure and LDL-Cholesterol Goals among Younger Adults with Type 2 Diabetes. *Sains Malaysiana*.
99. Wan, K. S., Hairi, N. N., Mustapha, F., Ismail, M., Mohd Yusoff, M. F., & Moy, F. M. (2022). Five-year LDL-cholesterol trend and its predictors among type 2 diabetes patients in an upper-middle-income country: a retrospective open cohort study. *PeerJ*.
100. Wan, K. S., Hairi, N. N., Mustapha, F. I., Mohd Yusof, K., Mohd Ali, Z., & Moy, F. M. (2021b). Predictors of glycosylated haemoglobin A1C trend among type 2 diabetes patients in a multi-ethnic country. *Scientific reports*.
101. Wan, K. S., Moy, F. M., Mustapha, F. I., Ismail, M., & Hairi, N. N. (2021c). Changes in body mass index, glycosylated hemoglobin A1C, blood pressure, and LDL-cholesterol among type 2 diabetes patients in Malaysia: A population-based longitudinal study. *Journal of diabetes*.
102. Wong, J. S. (2005). Proteinuria in diabetic patients in a primary health care setting in Sarawak. *Medical Journal of Malaysia*.
103. Wong, J. S., & Rahimah, N. (2004). Glycaemic control of diabetic patients in an urban primary health care setting in Sarawak: the Tanah Puteh Health Centre experience. *Medical Journal of Malaysia*.
104. Wong, J. S., Tan, F., & Lee, P. Y. (2007). The state of lipid control in patients with diabetes in a public health care centre. *Asia-Pacific journal of public health*.
105. Wong, W. L., Valliappan, V. N., Leong, M. C., Aminudin, S. N. A., Chew, S. C. J., & Cheong, A. T. (2020). Prevalence of microvascular complications in newly diagnosed type 2 diabetes mellitus in primary healthcare clinics. *Malaysian Journal of Medicine and Health Sciences*.
106. Yahya, N. S., Abdul, M. Z., & Che, D. A. Z. (2023a). PREVALENCE AND DETERMINANTS OF DIABETES DISTRESS AMONG TYPE 2 DIABETES MELLITUS (T2DM) WITH INSULIN THERAPY IN A PRIMARY HEALTH CARE CENTRE. *Journal of Health and Translational Medicine*.
107. Yahya, N. S., Awang, A., & Nordin, R. (2023b). ASSOCIATION BETWEEN INSULIN THERAPY ADHERENCE AND GLYCATED HAEMOGLOBIN (HBA1C) AMONG PATIENTS WITH TYPE 2 DIABETES MELLITUS ATTENDING A COMMUNITY HEALTH CLINIC. *Journal of Sustainability Science and Management*.
108. Yudin, Z. M., Yaacob, L. H., Hassan, N. B., Ismail, S. B., Draman, N., & Yusoff, S. S. M. (2017). Achievement of LDL Cholesterol Goal and Adherence to Statin by Diabetes Patients in Kelantan. *The Malaysian journal of medical sciences : MJMS*.
109. Zhu, T. H., Mooi, C. S., Shamsuddin, N. H., & Mooi, C. S. (2019). Diabetes empowerment scores among type 2 diabetes mellitus patients and its correlated factors: A cross-sectional study in a primary care setting in Malaysia. *World journal of diabetes*.
