# Supplementary material for: The quality of care for type 2 diabetes mellitus management in Malaysian primary health care settings: A scoping review of ABC (glycated haemoglobin A1c, blood pressure, and LDL-cholesterol)
Source: PLoS One. 2026 Jul 31;21(7):e0355227. doi: 10.1371/journal.pone.0355227 (PMC13426932; doi:10.1371/journal.pone.0355227)
Supplement: S5 Table — (DOCX) [file pone.0355227.s009.docx]

**S5 Table. Summary of funding sources received for the 109 included publications**

| **Funding source type** | **Number of publications, n (%)** |
| --- | --- |
| Non-sponsored | 1 (0.9%) |
| Not specified | 56 (51.4%) |
| Private-sponsored | 2 (1.8%) |
| Professional body sponsored | 2 (1.8%) |
| Public-sponsored | 48 (44.0%) |

For this scoping review, Ang SH received funding from the Ministry of Health Malaysia (Grant number: NMRR ID-24-00853-YHT (IIR)). The funder’s website is https://nmrr.gov.my. The sponsors or funders had no role in the study design, data collection and analysis, decision to publish, or preparation of the manuscript.
