# Supplementary material for: The quality of care for type 2 diabetes mellitus management in Malaysian primary health care settings: A scoping review of ABC (glycated haemoglobin A1c, blood pressure, and LDL-cholesterol)
Source: PLoS One. 2026 Jul 31;21(7):e0355227. doi: 10.1371/journal.pone.0355227 (PMC13426932; doi:10.1371/journal.pone.0355227)
Supplement: S6 Table — (DOCX) [file pone.0355227.s010.docx]

**S6 Table. Funding sources received for the 109 included publications**

| **Author (Year Published)** | **Funding source type** |
| --- | --- |
| (Ong et al., 2022) | Not specified |
| (Gunggu et al., 2016) | Not specified |
| (Lee et al., 2020) | Public-sponsored |
| (Naserrudin et al., 2022) | Not specified |
| (Husain et al., 2023) | Public-sponsored |
| (Jamaluddin & Mohamed Kamel, 2024) | Not specified |
| (Ab Rahman et al., 2022) | Public-sponsored |
| (Nordin et al., 2021) | Not specified |
| (Lim et al., 2019) | Public-sponsored |
| (Nasir et al., 2022) | Public-sponsored |
| (Chew et al., 2021) | Public-sponsored |
| (Syed Soffian et al., 2019) | Not specified |
| (Wan et al., 2022) | Not specified |
| (Chamhuri et al., 2022) | Not specified |
| (Lim et al., 2021) | Public-sponsored |
| (Lai et al., 2020) | Public-sponsored |
| (Abdullah et al., 2020) | Public-sponsored |
| (Sazlina et al., 2020) | Public-sponsored |
| (Dhillon et al., 2019) | Not specified |
| (Leelavathi et al., 2013) | Public-sponsored |
| (Zhu et al., 2019) | Public-sponsored |
| (Tharek et al., 2018) | Public-sponsored |
| (Goh et al., 2020) | Public-sponsored |
| (Chan, 2005) | Not specified |
| (Mahmood et al., 2016) | Not specified |
| (Kaur et al., 2013) | Not specified |
| (Ching et al., 2013) | Not specified |
| (B.H. Chew et al., 2015) | Public-sponsored |
| (Wong & Rahimah, 2004) | Not specified |
| (Chen et al., 2022) | Not specified |
| (Tan & Ng, 2023) | Non-sponsored |
| (Chin et al., 2023) | Not specified |
| (Yahya et al., 2023a) | Public-sponsored |
| (Yahya et al., 2023b) | Not specified |
| (Samat et al., 2024) | Not specified |
| (Bujang et al., 2021) | Public-sponsored |
| (Wan et al., 2021a) | Not specified |
| (Lee et al., 2022) | Professional body sponsored |
| (Nordin et al., 2020) | Not specified |
| (Rashid et al., 2020) | Not specified |
| (Lee et al., 2019) | Public-sponsored |
| (Rasid et al., 2020) | Public-sponsored |
| (Tan & Ismail, 2020) | Not specified |
| (Papo et al., 2019) | Public-sponsored |
| (Moy Foong & Yew Sheng, 2019) | Not specified |
| (Abdullah et al., 2017) | Not specified |
| (Jusoh et al., 2018) | Public-sponsored |
| (Chin et al., 2017) | Public-sponsored |
| (Mohd Aznan et al., 2018) | Public-sponsored |
| (Chew et al., 2017) | Public-sponsored |
| (Hashim et al., 2016) | Not specified |
| (W. L. Tan et al., 2015) | Not specified |
| (Azura et al., 2012) | Not specified |
| (K. C. Tan et al., 2015) | Not specified |
| (B. H. Chew et al., 2015b) | Public-sponsored |
| (Sazlina et al., 2010) | Not specified |
| (Chan et al., 2005) | Not specified |
| (Mallika et al., 2011) | Not specified |
| (Cheong et al., 2012) | Not specified |
| (Wong, 2005) | Not specified |
| (How et al., 2011) | Not specified |
| (Mafauzy, 2005) | Private-sponsored |
| (Mastura et al., 2007) | Public-sponsored |
| (Wong et al., 2007) | Not Specified |
| (Rabia & Khoo, 2007) | Not Specified |
| (S. F. Tan et al., 2015) | Public-sponsored |
| (Azmawati & Siti Norbayah, 2014) | Public-sponsored |
| (Azlina Wati et al., 2016) | Not Specified |
| (Nurjasmine Aida et al., 2018) | Not Specified |
| (Shibraumalisi et al., 2020) | Not Specified |
| (Swarna Nantha et al., 2017) | Public-sponsored |
| (Hassan et al., 2021) | Not Specified |
| (Chew et al., 2012b) | Public-sponsored |
| (Chew et al., 2012a) | Public-sponsored |
| (Tan et al., 2008) | Not Specified |
| (Alias et al., 2023) | Professional body sponsored |
| (Chew et al., 2013a) | Public-sponsored |
| (Chew et al., 2014) | Not Specified |
| (Chew et al., 2010) | Public-sponsored |
| (Azimah et al., 2010) | Not Specified |
| (Rohana et al., 2007) | Not Specified |
| (Yudin et al., 2017) | Public-sponsored |
| (Tajudin et al., 2020) | Not Specified |
| (Hui Ng et al., 2012) | Not Specified |
| (Singh et al., 2018) | Private-sponsored |
| (Norma et al., 2010) | Not Specified |
| (Wong et al., 2020) | Not Specified |
| (Ali et al., 2024) | Not specified |
| (Ang et al., 2024) | Public-sponsored |
| (Mastura et al., 2011) | Public-sponsored |
| (Lee et al., 2013) | Public-sponsored |
| (Wan et al., 2021b) | Public-sponsored |
| (Wan et al., 2021c) | Not Specified |
| (Nor Shazwani et al., 2010) | Not Specified |
| (Ahmad et al., 2014) | Public-sponsored |
| (Chew et al., 2011) | Not Specified |
| (Chew et al., 2018) | Public-sponsored |
| (Chew et al., 2013b) | Public-sponsored |
| (Lim et al., 2010) | Not Specified |
| (Ministry of Health Malaysia, 2013) | Public-sponsored |
| (Abdullah et al., 2019) | Public-sponsored |
| (Ministry of Health Malaysia, 2009) | Public-sponsored |
| (Ministry of Health Malaysia, 2010) | Public-sponsored |
| (How et al., 2012) | Not Specified |
| (Ministry of Health Malaysia, 2021) | Public-sponsored |
| (Mafauzy et al., 1999) | Not Specified |
| (B. H. Chew et al., 2015a) | Not Specified |
| (Ministry of Health Malaysia, 2020) | Public-sponsored |
| (Ministry of Health Malaysia, 2024) | Public-sponsored |

*Full citation of each study was provided below the S4 Table.
